# Computer-Aided Rapid Establishment of Fingerprint of Xiaojin Capsule by HPLC

**DOI:** 10.1155/2021/8858501

**Published:** 2021-01-16

**Authors:** Hui Jiang, Yuansheng Xiao, Xingya Xue, Hongli Jin, Yang Xiang, Yanfang Liu, Gaowa Jin

**Affiliations:** ^1^Key Lab of Separation Science for Analytical Chemistry, Dalian Institute of Chemical Physics, Chinese Academy of Sciences, Dalian 116023, China; ^2^University of Chinese Academy of Sciences, Beijing 100049, China; ^3^Jianmin Pharmaceutical Group Co., Ltd., Wuhan, Hubei 430000, China

## Abstract

Traditional Chinese medicine (TCM) formulas have a significant clinical efficacy, and the fingerprint technology has been widely accepted to fully reveal the quality of TCM. Whereas, it is a great challenge to establish the fingerprint chromatogram which can fully reflect every single herb material in a short time. In this study, we used Xiaojin capsule (XJC) as a case and developed a rapid fingerprint method based on increasing the column temperature and flow rate simultaneously combined with computer-aided. First, the elution gradient was optimized based on the retention parameters and peak shape parameters of the four linear gradients, and then, the column temperature and flow rate were increased simultaneously to shorten the analysis time. Next, the standard fingerprint chromatogram of XJC, which can reflect every herb material, was generated. Finally, quality markers were screened through unsupervised cluster analysis and supervised orthogonal partial least squares discrimination analysis. Combining computer-aided with increasing column temperature and flow rate simultaneously can develop the rapid method for establishing HPLC fingerprint of XJC, which can fully reflect every single herb material and provide comprehensive quality control. The strategy for establishing HPLC fingerprint of TCM formula could be applied to other traditional Chinese medicine formulas and herbal medicine.

## 1. Introduction

Traditional Chinese medicines (TCMs) have been used around the world for centuries in the prevention and treatment of human diseases. However, due to the complex material basis and the complex structure type of compounds of the TCMs, the uncontrollable quality is a bottleneck for its modernization and globalization all the time [1]. TCM formulas, containing two or more herbs, have a very significant clinic effect [2]. However, it is a great challenge to control the quality of TCM formulas, particularly those who contain ten or more herb materials.

The fingerprint, a spectrum, or chromatogram of the overall chemical composition can qualitatively and quantitatively reveal the changes in the pharmacodynamics of TCM. It can be obtained by modern separation and analysis methods such as chromatography or spectroscopy. Nowadays, fingerprint technology has been widely accepted to fully reveal the quality characteristics of TCMs all over the world, including the World Health Organization (WHO), the Chinese State Food and Drug Administration (SFDA), the Food and Drug Administration (FDA), and the European Medical Organization (EMA). Many methods have been proposed, including HPTLC, HPLC, GC, and CE for establishing characteristic fingerprints to identify TCM formula [3–5]. Among them, high-performance liquid chromatography (HPLC) has been widely used for establishing fingerprints of TCM owing to its high separation efficiency, good reproducibility, high precision, and easy operation. It has been reported that the chromatographic fingerprint can provide a comprehensive evaluation for TCM formulas [6, 7].

The complex components in TCM formula have a wide range of retention time and different retention behaviors in HPLC, so that it is a difficult task to establish the fingerprint of TCM formula in a short time. Several studies have established the HPLC fingerprints of TCM formula with a particularly long analysis time [8–10]. Longer analysis time means more solvent consumption and lower analysis efficiency. Gradient elution has been widely used in the analysis of TCM formula to improve separation efficiency [11]. Trial-and-error optimizations are frequently used to ﬁnd out the appropriate gradients, and they result in a long experimental cycle and low efficiency to establish the analytical method in yet another way. Optimization of the gradient elution conditions coupled with computer-aided allows for satisfactory separation without unnecessary experimentation. Optimization time will be saved, and optimization quality will be improved. To simplify and accelerate the optimization process, several computer simulation software packages, such as DryLab, Preopt-W, Osiris, Chrom, Sword, and PIOTR, have been widely used in reversed-phase high-performance liquid chromatography (RP-HPLC) [12–19]. Our laboratory developed a complex sample analysis software system (CSASS) to resolve the separation of complex samples [11]. The retention parameters and peak shape parameters can be calculated based on 3–5 linear gradient mobile phase conditions. Then, the simulated chromatogram under other gradient conditions can be exported, and the moved overlapping separation range mapping (OSRM) was drawn, so that the satisfactory separation condition of target peaks can be selected [20]. However, most reports referred to CSASS focus on the separation and analysis of herb materials or target compounds, such as Li et al. proposed a HPLC-DAD-ESI-MS/MS method for the analysis of iridoid glycosides (IGs) from *Hedyotis diffusa* Willd. through CSASS [21] and few studies referred to establishing fingerprints of TCM formula via CSASS.

Xiaojin capsule (XJC), a TCM formula originating from Xiaojin Dan, which was first recorded in Waike Zhengzhi Quansheng Ji by Hongxu Wang in the Qing Dynasty, has been recorded by China pharmacopoeia (version 2015). It is composed of artificial *Moschus* (Shexiang, SX), Faeces Trogopterori (Wulingzhi, WLZ), *Pheretima* (Dilong, DL), Momordicae Semen (Mubiezi, MBZ), Aconiti Kusnezoffii Radix Cocta (Caowu, CW), Angelicae Sinensis Radix (Danggui, DG), Liquidambaris Resina (Feng Xiang Zhi, FXZ), Olibanum (Ru Xiang, RX), *Myrrha* (Mo Yao, MY), and fragrant ink (Xiangmo, XM). The sources of these herb materials are complicated, including botanic, resinous, mineral, and creatural medicine. Xiaojin capsule and Xiaojin tablet are modified dosage forms of Xiaojin pill, and the following are collectively referred to as XJC. The three dosage forms were extensively used to treat multiple diseases, such as anti-inflammatory [22], hyperplasia of mammary gland [23], and prostate disease [24].

To develop a method for establishing HPLC fingerprints of TCM formula rapidly, we proposed to shorten analysis time through increasing the column temperature and flow rate simultaneously combined with computer-aided. Taking XJC as a case, the complex sample analysis software system (CSASS) was used to optimize the elution gradient. Then, the developed method was applied to analyze several batches of XJC samples and establish the standard fingerprint chromatogram. As a result, the standard fingerprint chromatogram can reflect every herb material in XJC and the differences of XJC samples from different manufacturers.

## 2. Experimental

### 2.1. Crude Drugs, Reference Standards, and Reagents

HPLC grade methanol was obtained from Meryer (Shanghai) Chemical Technology Co., Ltd., HPLC grade acetonitrile was purchased from Merck, the water was puriﬁed using a Milli-Q water puriﬁcation system (Bedford, MA, USA), and formic acid and phosphoric acid were purchased from J and K Scientific Ltd. (Beijing, China). A total of 36 batches of XJC samples were obtained from seven manufacturers (named A, B, C, D, E, F, and G), and their related messages are given in Table 1. The 10 herb materials were provided and authenticated by the manufacturer A.

### 2.2. Apparatus and Chromatographic Conditions

HPLC analysis was carried out by a Waters Alliance HPLC instrument (Waters Corporation, MA, USA), including the HPLC quaternary pump, an autosampler, a column oven, a binary solvent manager, and a PDA detector, connected to a Waters Empower 3 software. Separation was performed on a Waters-ACCHROM Tnature C18 column (250 mm ×4.6 mm i.d., 5 *µ*m, Waters, USA), and the column temperature was set at 40°C. On the basis of the eluent system with optimized ionization efficiency, the linear gradient consisted of A (acetonitrile) and B (0.2% (v/v) phosphoric acid in aqueous phase) with a flow rate of 1.5 mL•min^−1^. Four linear gradient program conditions were investigated in (i)–(iv) to obtain the optimized separation with the CSASS software. The optimized elution gradient was 0–40 min, 12%–98% A and 40–55 min, 98%-98% A, and the injection volume was 10 *μ*L.

- 0–120 min, 6%–98% A and 120–145 min, 98% A
- 0–90 min, 8%–98% A and 90–115 min, 98% A
- 0–60 min, 10%–98% A and 60–85 min, 98% A
- 0–30 min, 14%–98% A and 30–55 min, 98% A

### 2.3. Preparation of XJC Sample

In order to extract the effective components from XJC, the pretreatment of samples is necessary. In several of the studies, XJC samples were extracted by ultrasonic extraction with methanol as extraction solvent. Their research results show that this extraction method was effective [22, 25]. Therefore, in our research, the powders of XJC samples and ten herb materials were precisely weighed (2.0 g) and then ultrasonically extracted with 25 mL of methanol for 35 minutes below 30°C. After the extracting solution was agitated and cooled down, it was filtered through a 0.22 *μ*m microfiltration membrane. Then, the test solutions were obtained.

### 2.4. Methodology

#### 2.4.1. Measurement of Determination of Dead Time and Gradient Delay in the System

Dead time was measured with uracil on the Tnature C18 column. The mobile phase was isocratic elution with a composition of 50% methanol and 50% 0.1% (v/v) formic acid in aqueous phase. The absorbance wavelength was 254 nm.

Using A (water) and B (0.1% (v/v) acetone in aqueous phase) as mobile phase, a specific step gradient was designed to measure the gradient delay in the system. The C18 column was replaced by a capillary tube (25 cm, 0.001 in i.d), and the absorption spectrum at 265 nm was recorded. The elution gradient was 0–5 min, 95%-95% A; 5–25 min, 95%–5% A; and 25–30 min, 5%-5% A.

#### 2.4.2. Optimization of Elution Gradient by CSASS

**Figure 1:**
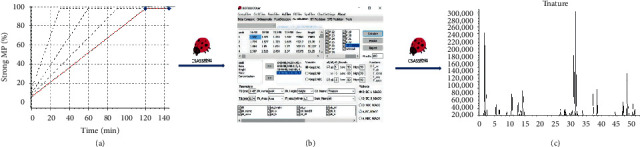
Flow diagram of optimizing elution conditions by CSASS.

The XJC sample was analyzed according to the four linear gradients in Section 2.2, and the absorption spectrum at 240 nm was recorded. The dead time, delay time, and four linear gradient analysis results were input into the CSASS software to optimize the elution gradient. The optimization process is shown in Figure 1.

#### 2.4.3. Precision, Repeatability, and Stability

The precision test was determined by the successive analyses of six injections of the same sample solution, respectively. Repeatability was determined by analyzing eight independent sample solutions extracted from the same batch of XJC samples. The stability was estimated by testing the same batch of sample solution stored at the same temperature for 0, 2, 4, 6, 8, 12, 24, 36, and 48 h, respectively. The XJC samples were analyzed, and the chromatograms were recorded.

#### 2.4.4. Generation of the Standard Fingerprint Chromatogram

The professional software Similarity Evaluation System for Chromatographic Fingerprint of Traditional Chinese Medicine (version 2004A) was used to generate the standard fingerprint chromatogram based on thirty-six batches of XJC samples. The similarities between different batches of XJC samples and the standard fingerprint chromatogram were evaluated.

#### 2.4.5. Identification of the Origin of Common Peaks

The XJC samples and ten herb materials solutions were analyzed by the method described in Section 2.3. The origin of common peaks could be identified based on the chromatogram and spectral absorption results acquired at 240 nm.

### 2.5. Data Analysis

Taking the common peak area in the standard fingerprint chromatogram as variables, the cluster analysis was performed based on the relative peak areas of those common characteristic peaks calculated by IBM SPSS Statistics 23. In this study, SIMCA-14.1 (Umetrics, Umea, Sweden) was used to carry out OPLS-DA for the standardized concentrations of all common peaks. Finally, the quality markers causing the differences between the XJC samples of different groups could be screened.

## 3. Results and Discussion

### 3.1. Optimization of Column Temperature and Mobile Phase

**Figure 2:**
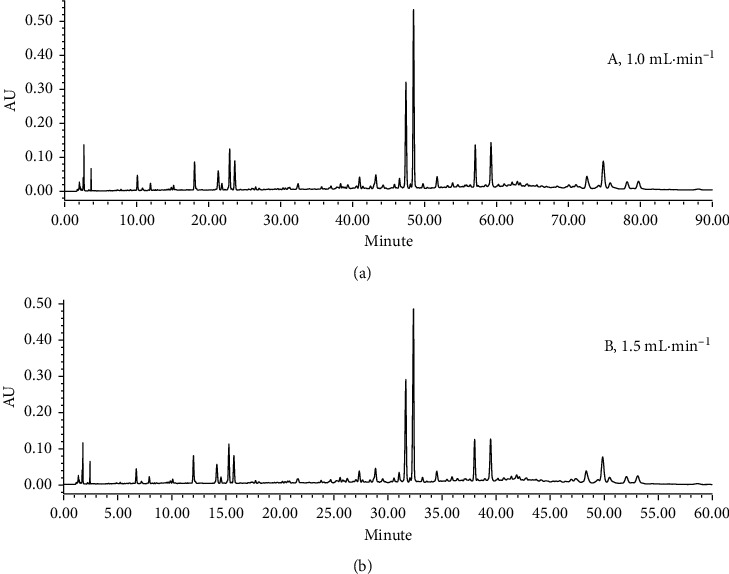
Chromatograms of XJC sample under two conditions. The analysis was performed on Waters Alliance HPLC instrument with the Tnature C18 column. The mobile phase was acetonitrile and 0.1% (v/v) phosphoric acid aqueous solution. The column temperature and flow rate were (a) 30°C, 1.0 mL•min^−1^; (b) 40°C, 1.5 mL•min^−1^. The detection wavelength was 240 nm. The elution gradient was 0–40 min, 12%–98% acetonitrile and 40–55 min, 98%-98% acetonitrile.

The effect of the column temperature (25, 30, 35, and 40°C) on the chromatographic peak separation was investigated, and the HPLC chromatograms with different column temperature were very similar, and 25°C or 30°C will be selected in general. It was worth mentioning that according to the van der Waals equation, the optimum flow rate of the column with an inner diameter of 4.6 mm is 1.0 mL•min^−1^ with room temperature as column temperature [26–29]. In this research, the analysis time was up to 90 minutes when the flow rate was 1.0 mL•min^−1^, and the result is shown in Figure 2(a). To shorten the analysis time, considering the higher column temperature can enhance the velocity of convection mass transfer, so that the decline in column efficiency of column could be reduced; we propose to increase the flow rate from 1.0 mL•min^−1^ to 1.5 mL•min^−1^, and 40°C was used as column temperature. The results under the higher flow rate are shown in Figure 2(b). In terms of peak shape and retention time of chromatographic peaks, the HPLC chromatograms under different column temperature and flow rate were consistent. The analysis time was reduced from 90 min to 60 min by increasing flow rate and column temperature. These results indicated that the method improves the separation analysis efficiency significantly.

Different mobile phase compositions were investigated, including acetonitrile-water, acetonitrile and 0.1% (v/v) phosphoric acid aqueous solution, and acetonitrile and 0.2% (v/v) phosphoric acid aqueous solution. The results demonstrated that the acetonitrile and 0.2% (v/v) phosphoric acid aqueous solution exhibited a satisfactory resolution, moderate retention time, and smooth baseline, which was adopted as the mobile phase in this study.

### 3.2. Optimization of Elution Gradient

**Figure 3:**
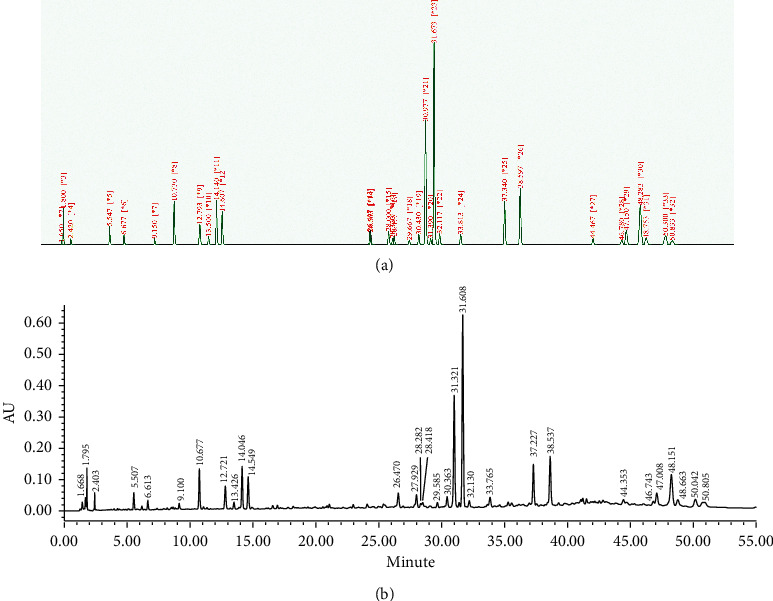
The simulated chromatogram (a) and the verification chromatogram (b) under the predicted elution gradient: 0–40 min, 12%–98% A and 40–55 min, 98%-98% A.

It is possible to predict retention behavior and optimize chromatographic separation [11] with CSASS. Based on the retention parameters and peak shape parameters obtained under four linear gradients, CSASS could give a simulated chromatogram for any elution conditions. The dead time (1.485 minutes), delay time (0.542 minutes), and retention parameters and peak shape parameters (Figure S1 and Tables S1) under four linear gradients were input into the CSASS software to predict the appropriate separation conditions, and then, the simulation conditions were experimentally performed and verified. The predicted optimal elution gradient was 0–40 min, 12%–98% A and 40–55 min, 98%–98% A; the simulated chromatogram is shown in Figure 3(a), and the experimental verification chromatogram is shown in Figure 3(b). Both the main and trace components in validated chromatogram have the same retention behavior with simulated chromatogram. Thus, the elution gradient was selected for analysis of the XJC samples.

### 3.3. Validation of the HPLC Fingerprint Method

The developed HPLC analytical approach was validated by estimating the precision, repeatability, and stability. The validation of precision, repeatability, and stability was performed on the relative standard deviation (RSD, %) of relative retention times (RRTs) and relative peak areas (RPAs) of the common peaks, and the peak with RT = 30.79 min was selected as the reference peak. The results for the precision, repeatability, and stability tests are shown in Table S3, respectively. In the precision test, the RSD values of the RRTs and RPAs were less than 0.20% and 4.14%. In the repeatability test, the RSD values of the RRTs and RPAs were less than 0.09% and 5.98%. Moreover, in the stability test, the RSD values of RRTs and RPAs were less than 0.21% and 6.30%. These results showed that the developed method is precise, accurate, and stable, and it is suitable to identify the fingerprint and twenty-five common peaks.

### 3.4. Generation of the Standard Fingerprint Chromatogram and Similarity Calculation

The HPLC fingerprint profiles of thirty-six batches of XJC samples acquired from seven manufacturers were obtained (Figure S2). According to the similarity calculation results of samples from the same manufacturer, the samples from A had a higher stability and consistency than other manufacturers. Therefore, XJC samples from A were fitted to generate the standard fingerprint chromatogram. The standard fingerprint chromatogram generated by the Similarity Evaluation System for Chromatographic Fingerprint of Traditional Chinese Medicine (version 2004A). And then, the similarities between different batches of XJC samples' fingerprints and the standard fingerprint chromatogram were calculated (Table S2). The result showed that the similarities of XJC samples from both different and same manufacturers had a big difference. Therefore, it is necessary to control the source and quality of single herb material and the process to maintain the stability and consistency of XJC.

### 3.5. Identification of the Origin of Common Peaks

**Figure 4:**
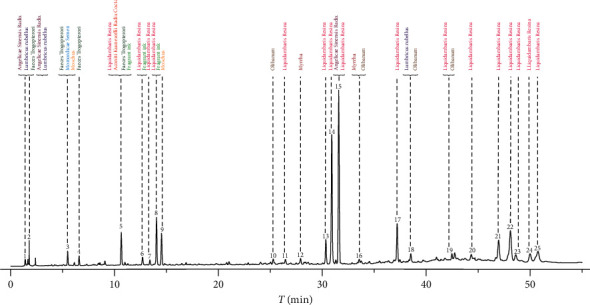
The origin of common peaks in the standard fingerprint chromatogram of XJC samples.

In this research, twenty-five common peaks were assigned based on the comparison of their retention time and absorption spectra with the herb material solutions (Figures S3 and S4). The standard fingerprint chromatogram contained twenty-five common peaks as shown in Figure 4. Peaks 4 was the characteristic peak from WLZ, and the extracts of WLZ can inhibit the proliferation of lymphocyte U937 in vitro [30]. Peaks 7, 11, 13, 14, 17, 20, 21, 22, 23, 24, and 25 were the characteristic peaks from FXZ, and FXZ could activate blood and relieve pain in traditional Chinese medicine theory. Peak 9 was the characteristic peak from SX, which had antithrombin activity [31]; moreover, SX for tumor growth in nude mice has a certain antitumor effect [32]. Peak 10 was the characteristic peak from RX. According to modern pharmacology research, RX extracts possess anti-inflammatory [33], antiasthma [34], antioxidant bioactivities [35], and antiulcer [36]. Peak 12 was a characteristic peak from MY. Prior to the discovery of morphine, MY was a commonly used analgesic, which had many activities such as anti-inflammatory [37], antibacterial [38], anthelmintic [39], and anticancer [40]. Peaks 1, 2, 3, 5, 6, 8, 15, 16, 18, and 19 were from two or more herb materials. Finally, the origins of twenty-five common peaks were confirmed, and ten herb materials were all reflected in the standard fingerprint chromatography.

### 3.6. Chemical Pattern Recognition

#### 3.6.1. Clustering Analysis (CA)

**Figure 5:**
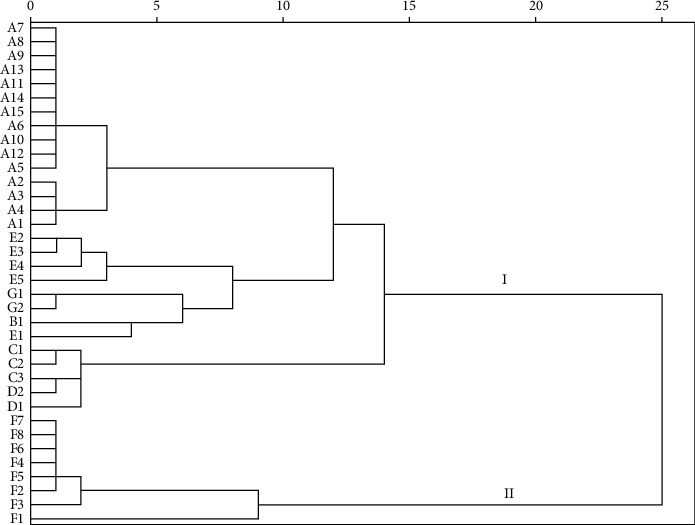
The CA results of different batches of XJC samples.

The cluster analysis showed that thirty-six batches of XJC were sorted into two clusters by selecting 15 as distance degree (Figure 5). The samples in cluster I were from manufactures A, B, C, D, E, and G, and the cluster II come from F. It indicated that the samples from F had a difference with the other manufactures. The differences may be caused by both the source of the herb materials and the processing of XJC samples.

#### 3.6.2. Orthogonal Partial Least Squares Discrimination Analysis (OPLS-DA)

**Figure 6:**
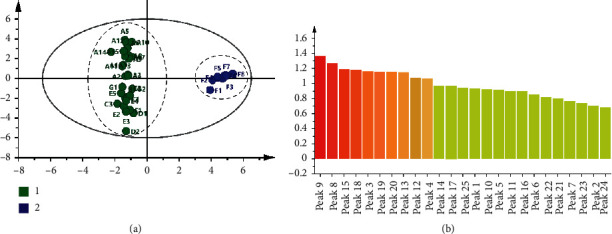
(a) The OPLS-DA results of different batches of XJC samples; (b) the VIP values of 25 common peaks in the OPLS-DA model.

Orthogonal partial least squares discrimination analysis (OPLS-DA) was carried out based on the CA, and the results are shown in Figure 6(a). The model principal component regression coefficients were R^2^_X_ = 0.911, R^2^_Y_ = 0.983, and *Q*^2^ = 0.881 > 0.5. In the prediction model, Q^2^ was greater than 0.5. This value indicated that the OPLS-DA prediction model was effective, and two groups of XJC samples were distinguished well. The results are consistent with the CA. Both the CA result and the OPLS-DA result indicated that the samples from F had a big difference with other manufacturers.

The VIP values of variables in the OPLS-DA model represent the roles they played in classification, and the bigger the value means more important. Thus, the VIP values of twenty-five common peaks were extracted and arranged from large to small in the OPLS-DA model (Figure 6(b)). The VIP values of the peaks 9, 8, 15, 18, 3, 19, 20, 13, 12, and 4 were greater than 1; it means that these common peaks played more important roles in distinguishing the two groups of XJC samples. Among them, peak 9 originated from SX, peak 8 originated from FXZ and XM, peak 15 originated from DG and FXZ, peak 18 originated from MY, DL, and RX, peak 3 originated from WLZ, SX, and MBZ, peak 19 originated from FXZ and RX, peak 20 and peak 13 originated from FXZ, peak 12 originated from MY, and peak 4 originated from WLZ. Overall, the above 10 peaks originated from nine herb materials making up XJC except CW. The content of diester-type alkaloids from CW was controlled strictly in the Chinese pharmacopoeia of the 2015 edition. Above all, the 10 peaks with VIP value > 1 could be selected as the markers to evaluate the quality consistency of XJC.

## 4. Conclusions

In this study, we developed a fast method for the establishment of the HPLC fingerprint of TCM formula by increasing the flow rate and column temperature simultaneously combined with computer-aided. Taking XJC as an example, the established method can complete the chromatographic analysis of XJC in 55 minutes. After that, the fast-developing HPLC method was used to analyze 36 batches of XJC samples, and the standard fingerprint chromatogram was generated. In the standard fingerprint chromatogram, there were twenty-five common peaks derived from the ten herb materials. What is more, thirty-six batches of XJC samples were divided into two groups, and a total of ten quality markers were screened out through CA and OPLS-DA. Combining simultaneous increase of column temperature and flow rate with computer-aided can quickly establish HPLC fingerprints of TCM formula, and it can improve the efficiency of separation and analysis significantly. At the same time, the established fingerprints can provide comprehensive quality control for TCM formula. The proposed method for establishing HPLC fingerprint could be applied to other traditional Chinese medicines and herbal medicine.

## Figures and Tables

**Table 1:** Source and serial number of each passel sample.

Serial no.	Item no.	Batch no.	Serial no.	Item no.	Batch no.
S1	A1	181173	S19	C3	1706002Z
S2	A2	181174	S20	D1	170401
S3	A3	181175	S21	D2	170602
S4	A4	181176	S22	E1	17010002
S5	A5	181177	S23	E2	17080011
S6	A6	181178	S24	E3	17110013
S7	A7	181179	S25	E4	17120015
S8	A8	181280	S26	E5	17120016
S9	A9	181281	S27	F1	170804
S10	A10	181282	S28	F2	171002
S11	A11	181283	S29	F3	171004
S12	A12	181284	S30	F4	171005
S13	A13	181285	S31	F5	171104
S14	A14	181286	S32	F6	171204
S16	A15	181287	S33	F7	171206
S16	B1	171106	S34	F8	171208
S17	C1	1703002Z	S35	G1	17040016
S18	C2	1703003Z	S36	G2	17040018

## Data Availability

The data used to support the findings of this study are included within the supplementary information file.

## Abbreviations

TCM: Traditional Chinese medicine
XJC: Xiaojin capsule
CSASS: Complex sample analysis software system
CA: Cluster analysis
OPLS-DA: Orthogonal partial least squares discrimination analysis
OSRM: Overlapping separation ranging mapping
SX: Moschus (She xiang)
WLZ: Faeces Trogopterori (Wu ling zhi)
DL: Pheretima (Di long)
MBZ: Momordicae Semen (Mu bie zi)
CW: Aconiti Kusnezoffii Radix Cocta (Cao wu)
DG: Angelicae Sinensis Radix (Dang gui)
FXZ: Liquidambaris Resina (Feng Xiang Zhi)
RX: Olibanum (Ru Xiang)
MY: Myrrha (Mo Yao)
XM: Fragrant ink (Xiang mo).
