# Supplementary material for: Computer-Aided Rapid Establishment of Fingerprint of Xiaojin Capsule by HPLC
Source: Int J Anal Chem. 2021 Jan 16;2021:8858501. doi: 10.1155/2021/8858501 (PMC7826213; doi:10.1155/2021/8858501)
Supplement: Supplementary Materials — Supplementary data related to this article can be found in supplementary information. [file 8858501.f1.docx]

**Computer-aided rapid establishment of** **fingerprint of**

**Xiaojin Capsule by HPLC**

Hui Jiang ^a^, Yuansheng Xiao ^a^, Xingya Xue ^a^, Hongli Jin ^a^, Yang Xiang ^b^,

Yanfang Liu ^a, *^, Gaowa Jin ^a, *^

^a^ Key Lab of Separation Science for Analytical Chemistry, Dalian Institute of Chemical Physics, Chinese Academy of Sciences, Dalian 116023, China

^b^ Jianmin Pharmaceutical Group Co., Ltd., Wuhan, Hubei 430000, China.

Fax: +86-411-84379539 Tel.: +86-411-84379519

Email: [liuyanfang@dicp.ac.cn](mailto:liuyanfang@dicp.ac.cn) [jingw@dicp.ac.cn](mailto:jingw@dicp.ac.cn)

Supporting information

- **Optimization of elution gradient**

Figure S1(A)-(D) are chromatograms of XJC sample under four gradient mobile phase conditions. We numbered peaks based on the retention time and absorption spectrum under four linear gradients as much as possible.

The dead time (1.485 minutes), delay time (0.542 minutes) and retention parameters and peak shape parameters under four linear gradients in Tables S1 were input into the CSASS software to predict the appropriate separation conditions.


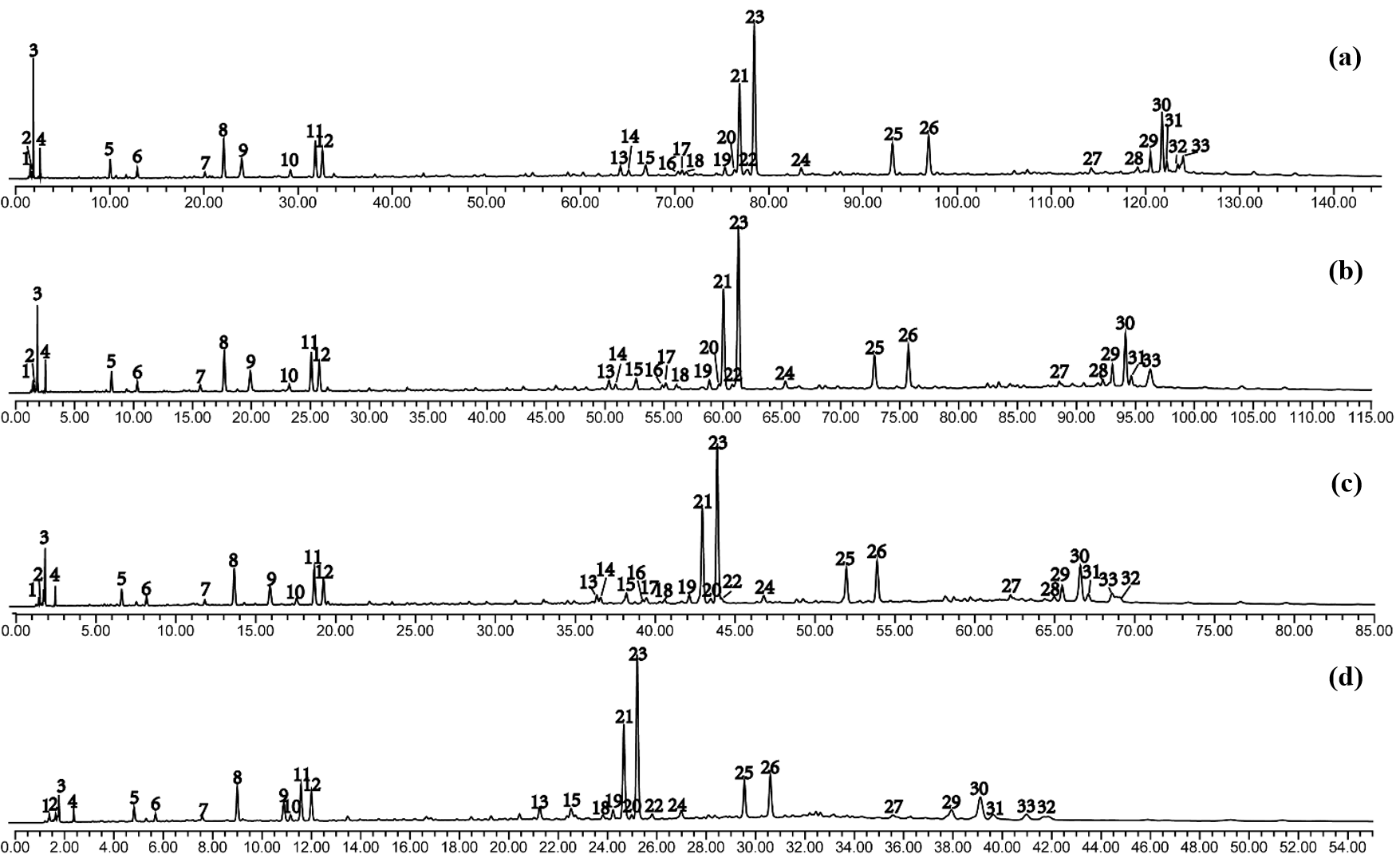


**Fig. S1** Chromatograms of XJC sample under four gradient mobile phase conditions. The mobile phase was (A) acetonitrile, (B) 0.2% (v/v) phosphoric acid in aqueous phase. Gradient conditions were (a) 0-120 min, 6%-98%A, 120-145 min, 98%A; (b) 0-90 min, 8%-98%A, 90-115 min, 98%A; (c) 0-60 min, 10%-98%A, 60-85 min, 98%A; (d) 0-30 min, 14%-98%A, 30-55min, 98%A.

**Table S1.** Retention parameters and peak shape parameters of

four linear gradient mobile phase conditions

| peak | T6-98 | T8-98 | T10-98 | T14-98 | Area | Height |
| --- | --- | --- | --- | --- | --- | --- |
| 1 | 1.532 | 1.473 | 1.433 | 1.382 | 99861 | 24853 |
| 2 | 1.734 | 1.577 | 1.723 | 1.635 | 47217 | 13170 |
| 3 | 1.884 | 1.85 | 1.821 | 1.77 | 302111 | 211127 |
| 4 | 2.597 | 2.515 | 2.458 | 2.37 | 85375 | 53125 |
| 5 | 10.049 | 8.126 | 6.61 | 4.816 | 266387 | 33217 |
| 6 | 12.924 | 10.316 | 8.168 | 5.682 | 151712 | 16561 |
| 7 | 20.093 | 15.658 | 11.81 | 7.578 | 94575 | 9128 |
| 8 | 22.098 | 17.694 | 13.655 | 8.998 | 744589 | 66613 |
| 9 | 24.015 | 19.908 | 15.894 | 10.883 | 511909 | 32249 |
| 10 | 29.185 | 23.212 | 17.552 | 11.157 | 169741 | 12795 |
| 11 | 31.831 | 25.078 | 18.664 | 11.578 | 759145 | 63909 |
| 12 | 32.58 | 25.752 | 19.241 | 11.999 | 591096 | 47914 |
| 13 | 64.229 | 50.34 | 36.346 | 21.253 | 282104 | 16334 |
| 14 | 65.1 | 50.889 | 36.579 | 21.253 | 154630 | 9896 |
| 15 | 66.908 | 52.645 | 38.194 | 22.519 | 336595 | 18004 |
| 16 | 70.358 | 54.856 | 39.213 | 0 | 119358 | 7183 |
| 17 | 70.778 | 55.152 | 39.444 | 0 | 154073 | 9309 |
| 18 | 71.235 | 56.057 | 40.587 | 23.812 | 101427 | 6335 |
| 19 | 75.333 | 58.858 | 42.13 | 24.224 | 220413 | 14253 |
| 20 | 76.338 | 59.68 | 43.479 | 24.975 | 183362 | 9559 |
| 21 | 76.88 | 60.049 | 42.949 | 24.659 | 2490160 | 159862 |
| 22 | 77.728 | 60.786 | 44.145 | 25.806 | 253717 | 9880 |
| 23 | 78.445 | 61.316 | 43.878 | 25.203 | 4095200 | 264382 |
| 24 | 83.425 | 65.3 | 46.789 | 26.986 | 225012 | 12213 |
| 25 | 93.12 | 72.858 | 51.956 | 29.546 | 993575 | 54849 |
| 26 | 96.964 | 75.739 | 53.881 | 30.596 | 1208602 | 67083 |
| 27 | 114.217 | 88.53 | 62.229 | 35.566 | 196794 | 9777 |
| 28 | 119.17 | 92.234 | 64.967 | 0 | 239086 | 10979 |
| 29 | 120.52 | 93.044 | 65.464 | 37.935 | 496206 | 35788 |
| 30 | 121.757 | 94.155 | 66.6 | 39.103 | 1532420 | 107758 |
| 31 | 122.257 | 94.648 | 67.099 | 39.624 | 312009 | 21962 |
| 32 | 123.424 | 0 | 69.064 | 41.683 | 256321 | 16118 |
| 33 | 123.984 | 96.257 | 68.586 | 40.973 | 740706 | 29419 |

- **Generation of the standard fingerprint chromatogram and similarity calculation**

Different batches of XJC samples were analyzed and XJC samples from manufacture A were fitted to generate the standard fingerprint chromatogram. Table S2 showed the similarities between different batches XJC samples’ fingerprints and the standard fingerprint chromatogram.

**Table S2.** The similarities of 36 batches XJC samples’ fingerprint

compared with the standard fingerprint chromatogram

| Serial No. | Item No. | Batch No. | Similarity |
| --- | --- | --- | --- |
| S1 | A1 | 181173 | 0.925 |
| S2 | A2 | 181174 | 0.935 |
| S3 | A3 | 181175 | 0.951 |
| S4 | A4 | 181176 | 0.943 |
| S5 | A5 | 181177 | 0.976 |
| S6 | A6 | 181178 | 0.978 |
| S7 | A7 | 181179 | 1 |
| S8 | A8 | 181280 | 0.985 |
| S9 | A9 | 181281 | 0.996 |
| S10 | A10 | 181282 | 0.987 |
| S11 | A11 | 181283 | 0.999 |
| S12 | A12 | 181284 | 0.978 |
| S13 | A13 | 181285 | 0.984 |
| S14 | A14 | 181286 | 0.999 |
| S15 | A15 | 181287 | 0.981 |
| S16 | B1 | 171106 | 0.835 |
| S17 | C1 | 1703002Z | 0.797 |
| S18 | C2 | 1703003Z | 0.796 |
| S19 | C3 | 1706002Z | 0.789 |
| S20 | D1 | 170401 | 0.765 |
| S21 | D2 | 170602 | 0.736 |
| S22 | E1 | 17010002 | 0.893 |
| S23 | E2 | 17080011 | 0.708 |
| S24 | E3 | 17110013 | 0.754 |
| S25 | E4 | 17120015 | 0.667 |
| S26 | E5 | 17120016 | 0.743 |
| S27 | F1 | 170804 | 0.479 |
| S28 | F2 | 171002 | 0.365 |
| S29 | F3 | 171004 | 0.429 |
| S30 | F4 | 171005 | 0.318 |
| S31 | F5 | 171104 | 0.334 |
| S32 | F6 | 171204 | 0.337 |
| S33 | F7 | 171206 | 0.366 |
| S34 | F8 | 171208 | 0.335 |
| S35 | G1 | 17040016 | 0.784 |
| S36 | G2 | 17040018 | 0.740 |

- **Validation of the HPLC fingerprint method**

The validation of precision, repeatability and stability was performed on the relative standard deviation (RSD, %) of relative retention times (RRTs) and relative peak area (RPA) of the common peaks and the peak with RT=30.79 min was selected as the reference peak. Results for the precision, repeatability and stability tests were shown in Table S3, respectively.

**Table S3.** The precision, repeatability and stability tests of the common peaks in the standard fingerprint chromatogram

| Peak no. | Precision | | Repeatability | | | Stability | |
| --- | --- | --- | --- | --- | --- | --- | --- |
|  | RRT (RSD, %) | RPA (RSD, %) | | RRT (RSD, %) | RPA (RSD, %) | RRT (RSD, %) | RPA (RSD, %) |
| 1 | 0.199 | 1.230 | | 0.055 | 3.723 | 0.189 | 1.335 |
| 2 | 0.053 | 0.863 | | 0.086 | 3.292 | 0.132 | 1.821 |
| 3 | 0.065 | 0.728 | | 0.071 | 4.363 | 0.212 | 0.964 |
| 4 | 0.060 | 0.351 | | 0.075 | 3.478 | 0.202 | 0.832 |
| 5 | 0.052 | 0.491 | | 0.075 | 3.416 | 0.126 | 1.028 |
| 6 | 0.045 | 2.410 | | 0.065 | 2.720 | 0.091 | 3.510 |
| 7 | 0.050 | 0.562 | | 0.070 | 2.108 | 0.099 | 1.312 |
| 8 | 0.051 | 0.683 | | 0.062 | 2.382 | 0.089 | 0.787 |
| 9 | 0.052 | 0.454 | | 0.064 | 2.872 | 0.091 | 0.511 |
| 10 | 0.028 | 1.230 | | 0.038 | 2.300 | 0.047 | 2.699 |
| 11 | 0.024 | 2.630 | | 0.027 | 5.981 | 0.040 | 4.694 |
| 12 | 0.021 | 2.377 | | 0.027 | 1.852 | 0.037 | 4.174 |
| 13 | 0.017 | 0.293 | | 0.019 | 2.593 | 0.028 | 0.736 |
| 14 | 0.015 | 0.301 | | 0.017 | 2.707 | 0.024 | 0.750 |
| 15 | 0.014 | 0.406 | | 0.015 | 2.619 | 0.023 | 0.861 |
| 16 | 0.014 | 1.115 | | 0.015 | 2.522 | 0.024 | 1.705 |
| 17 | 0.005 | 4.144 | | 0.007 | 5.466 | 0.010 | 6.304 |
| 18 | 0.000 | 0.000 | | 0.000 | 0.000 | 0.000 | 0.000 |
| 19 | 0.011 | 0.466 | | 0.027 | 2.648 | 0.031 | 4.788 |
| 20 | 0.040 | 0.380 | | 0.034 | 2.928 | 0.065 | 1.968 |
| 21 | 0.013 | 0.392 | | 0.016 | 3.021 | 0.015 | 0.813 |
| 22 | 0.018 | 0.470 | | 0.020 | 3.097 | 0.018 | 0.697 |
| 23 | 0.020 | 0.566 | | 0.024 | 3.566 | 0.020 | 1.011 |
| 24 | 0.025 | 1.013 | | 0.033 | 4.039 | 0.027 | 1.566 |
| 25 | 0.028 | 1.427 | | 0.037 | 2.887 | 0.029 | 2.180 |

- **The HPLC fingerprints of 36 batches of XJC samples**

The HPLC fingerprints of 36 batches of XJC samples acquired from seven manufacturers were analyzed by established method.


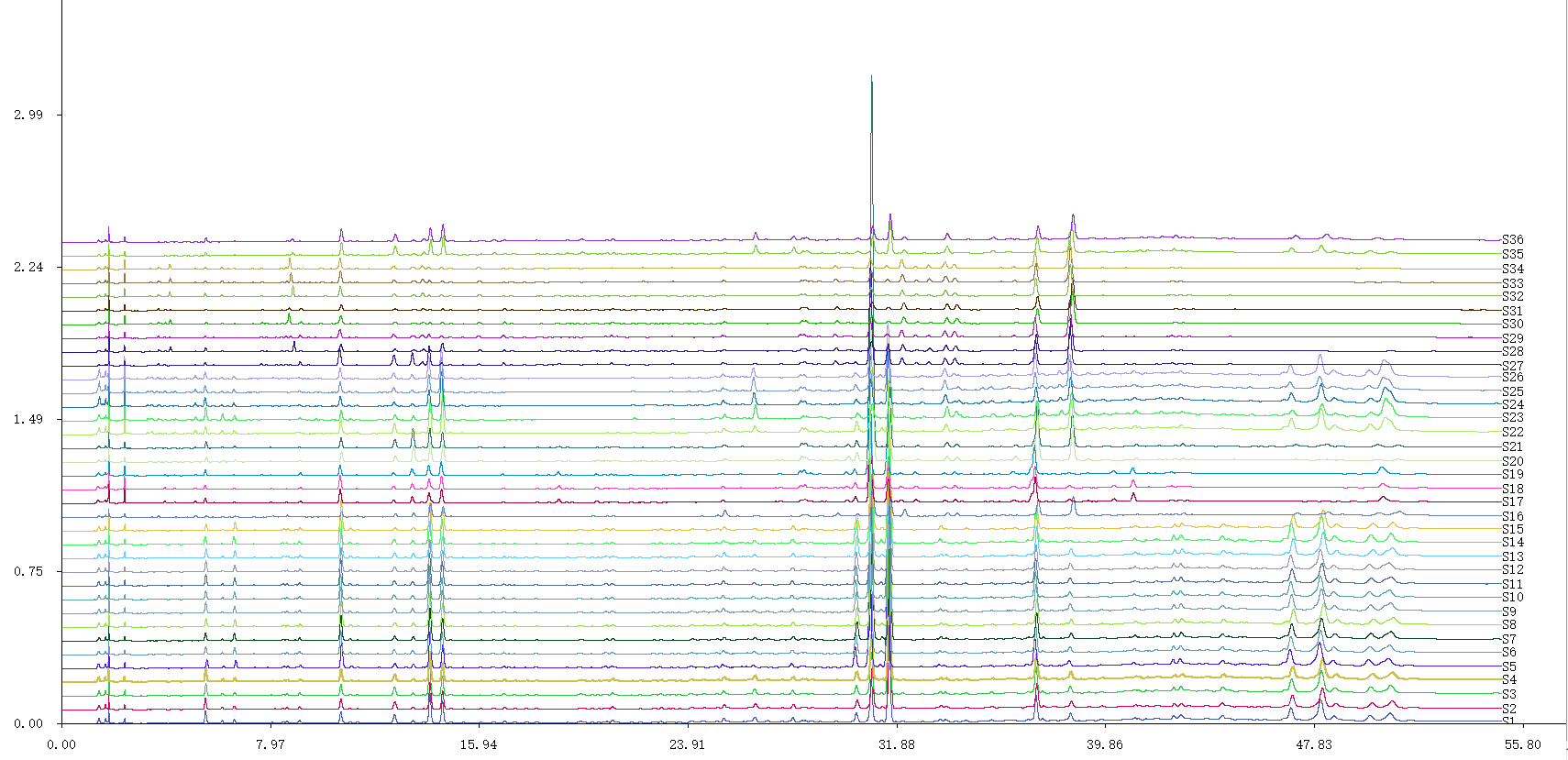


**Fig. S2** The HPLC fingerprints of 36 batches of XJC samples. The mobile phase was (A) acetonitrile, (B) 0.2% (v/v) phosphoric acid in aqueous phase. Gradient condition was 0-40 min, 12%-98%A; 40-55 min, 98%-98%A. The ﬂow rate was 1.5 mL•min^-1^ and the column temperature was maintained at 40℃.

- **The HPLC fingerprints of ten herb materials and identification of the origin of common peaks in standard fingerprint chromatogram**

In this research, twenty-five common peaks were assigned based on the comparison of their retention time and absorption spectra with the herb material solutions (figure S3).


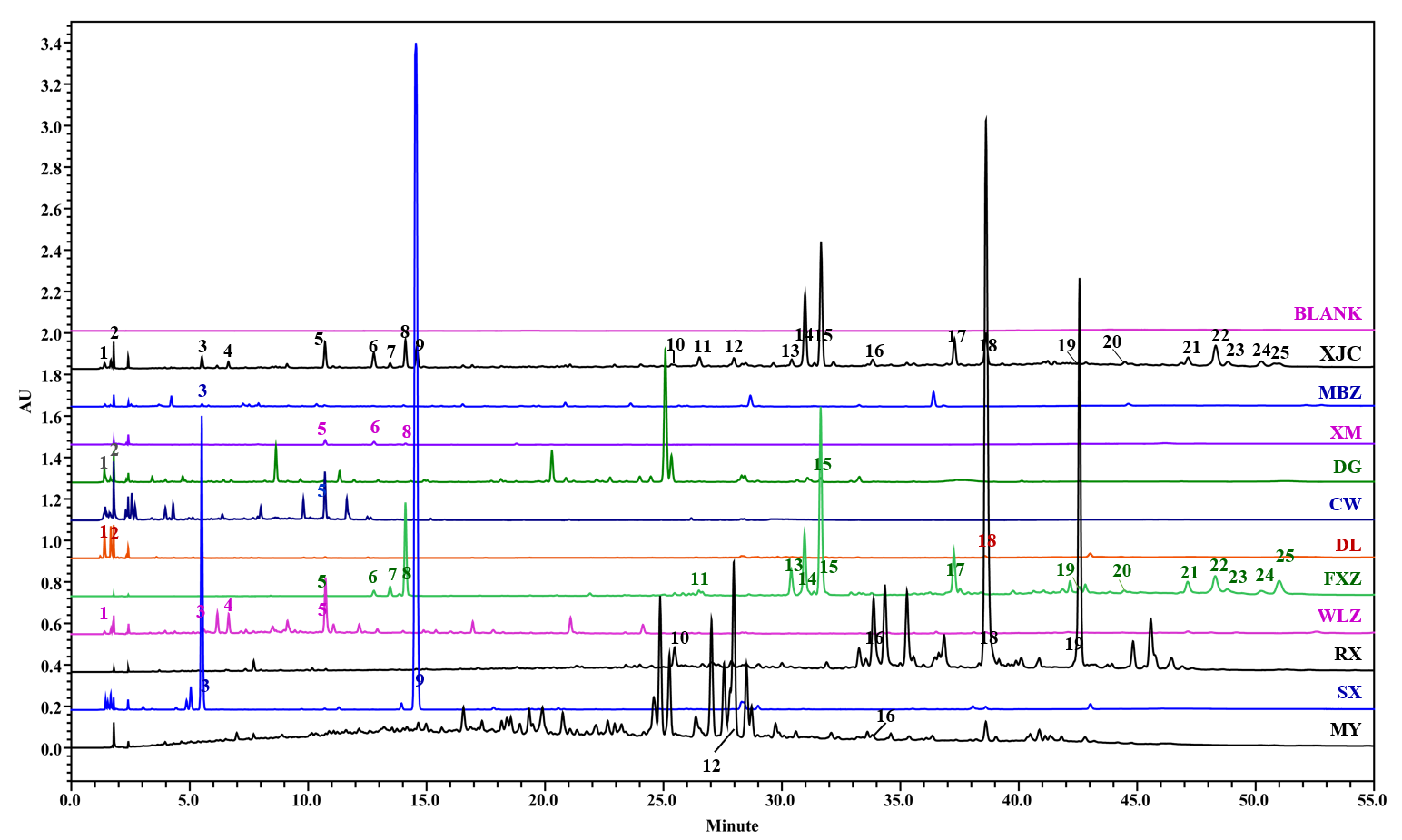


**Fig. S3** The HPLC fingerprints of ten herb materials and XJC samples.

- **The retention time and absorption spectra of common peaks in standard fingerprint chromatogram and ten herb materials**

Peak 1:


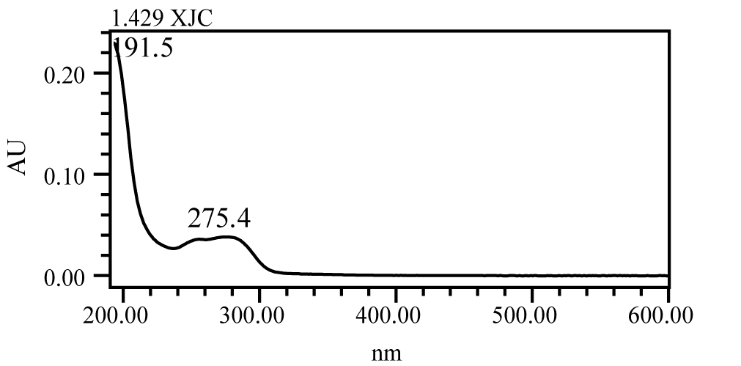

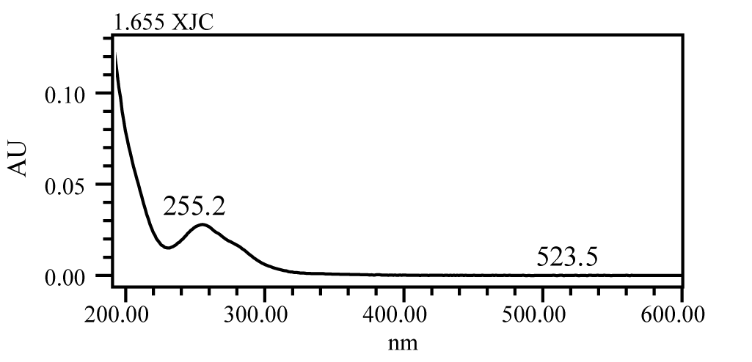

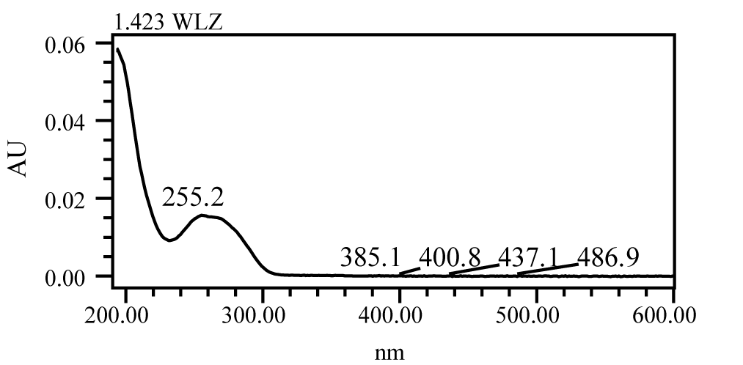

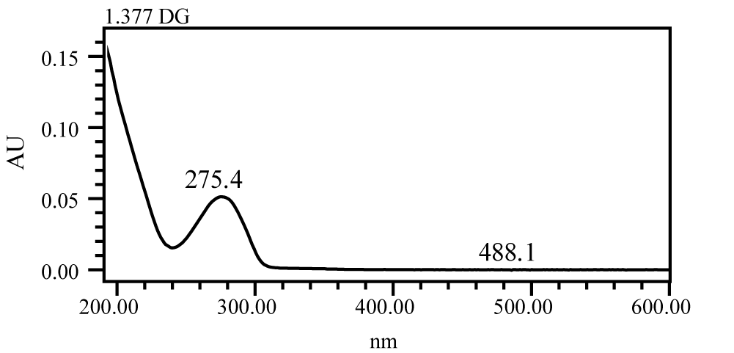

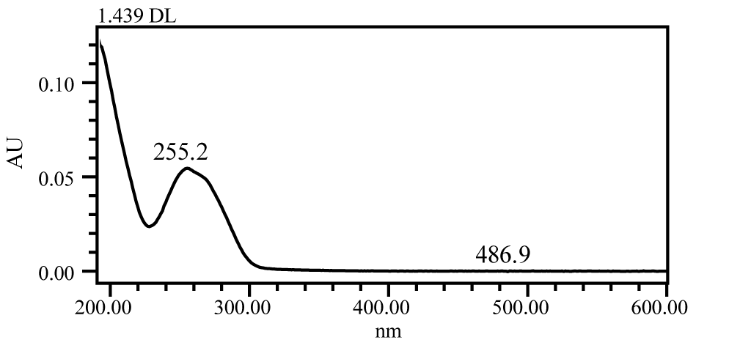


Peak 2:


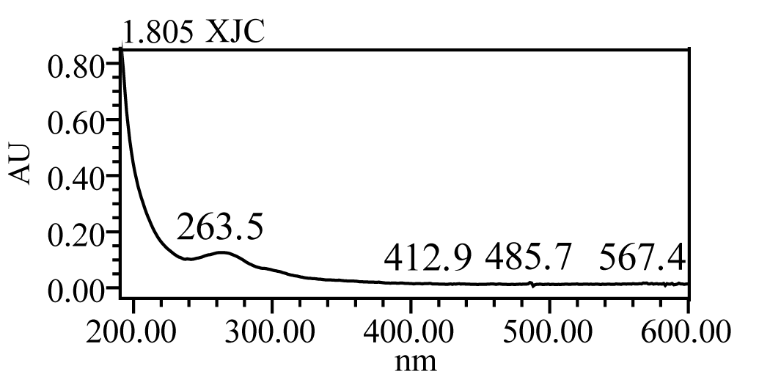


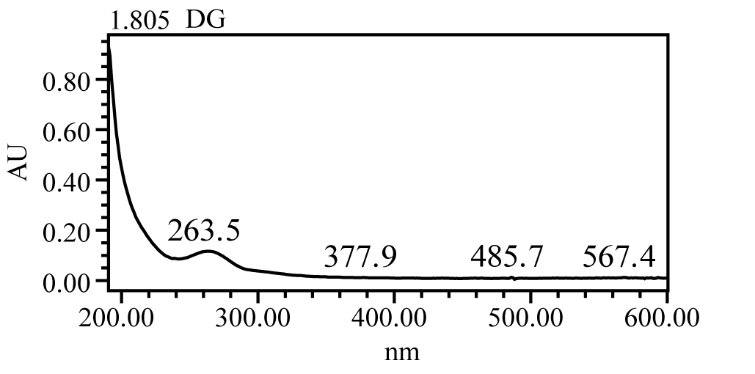


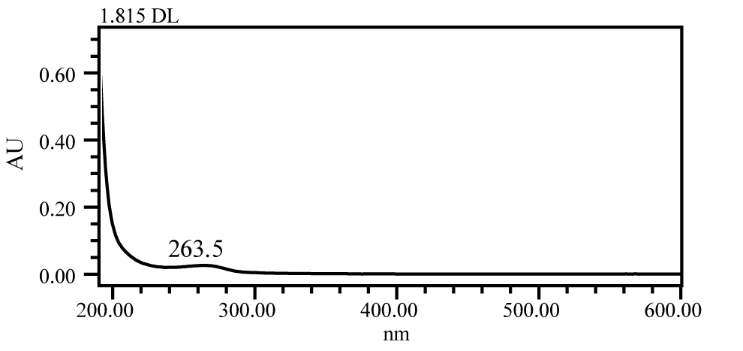


Peak 3:


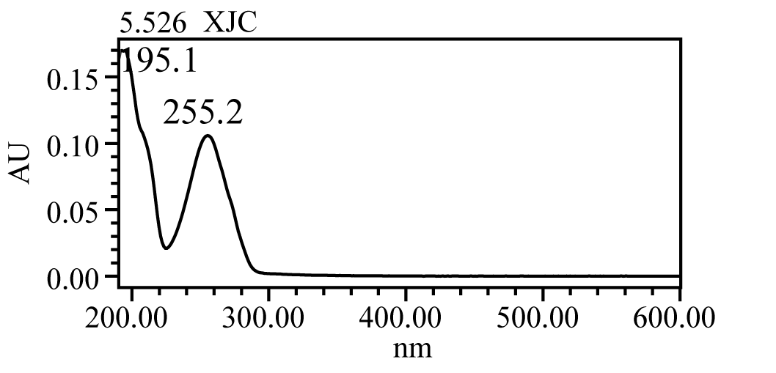


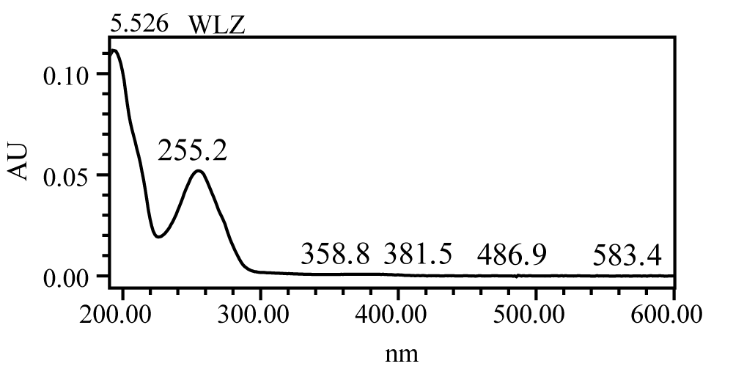


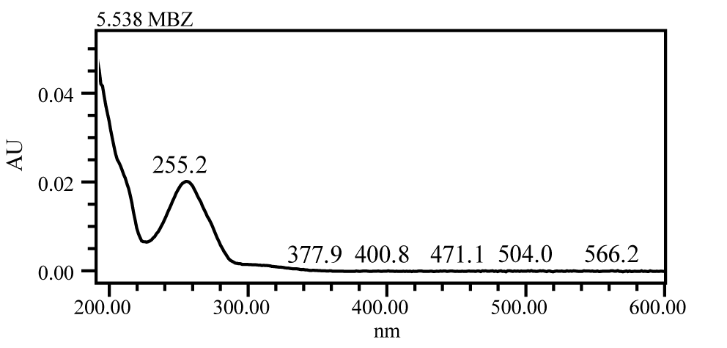


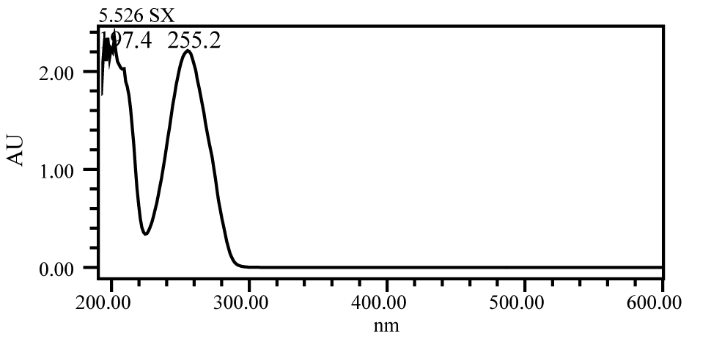


Peak 4:


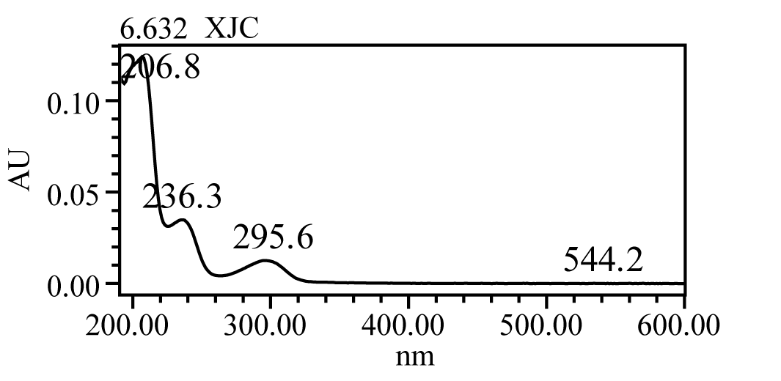


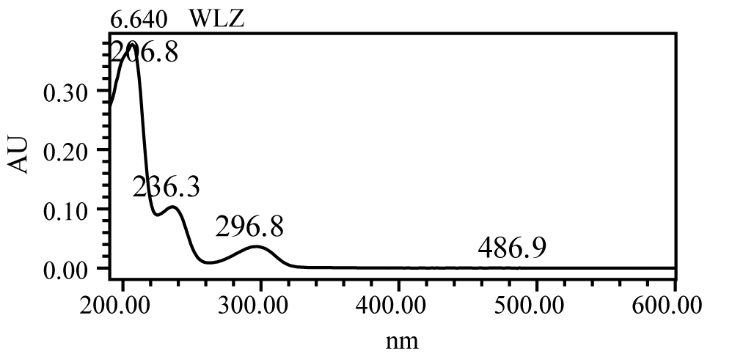


Peak 5:


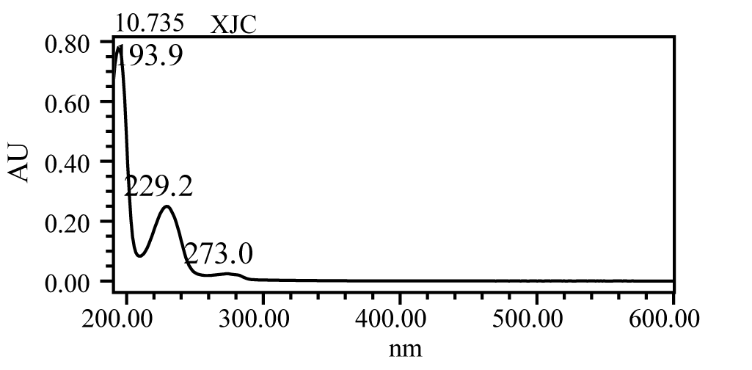

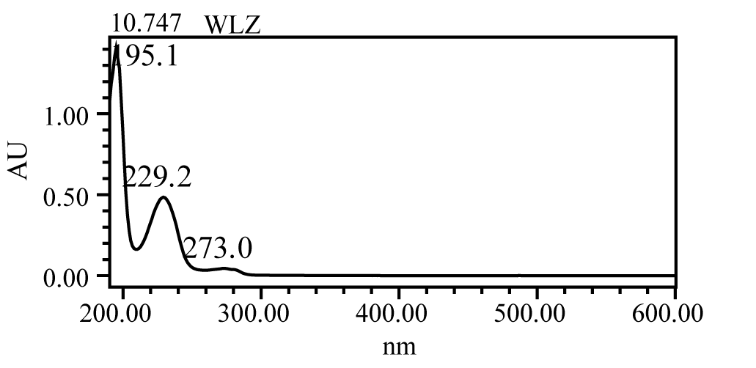

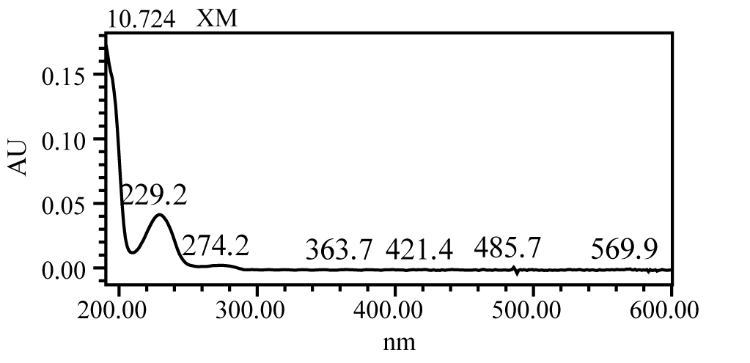

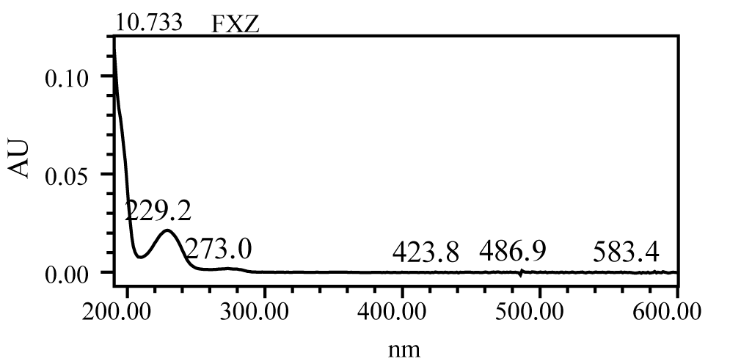

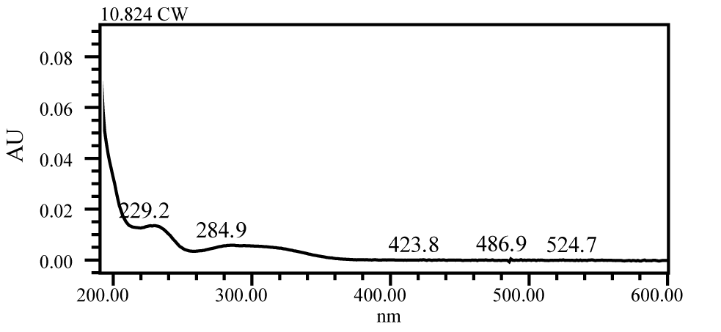


Peak 6:


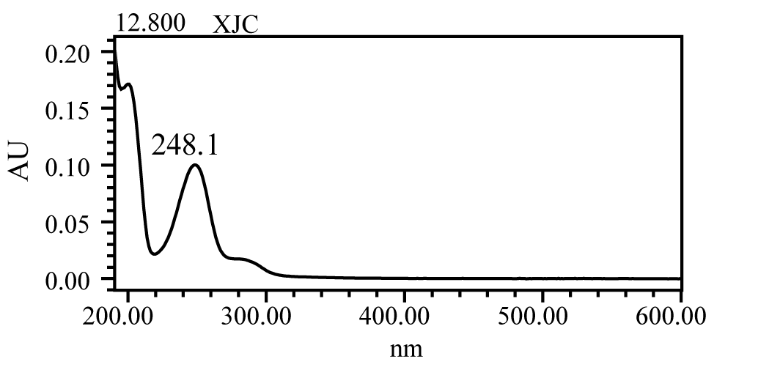


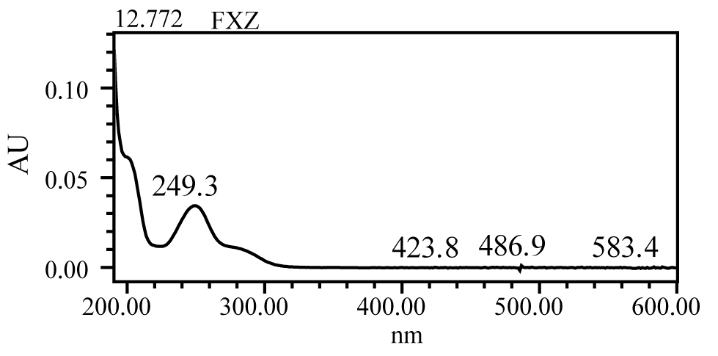


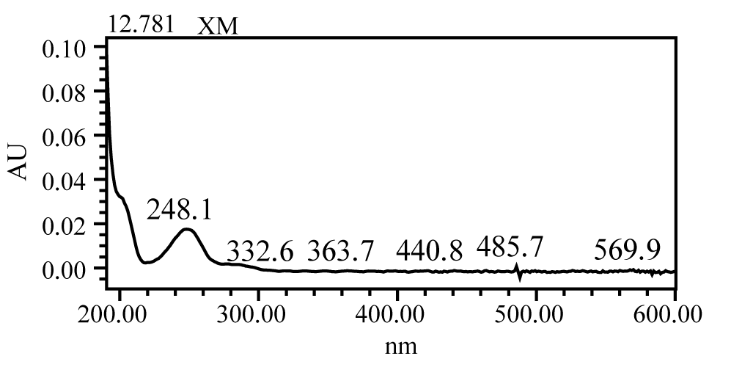


Peak 7:


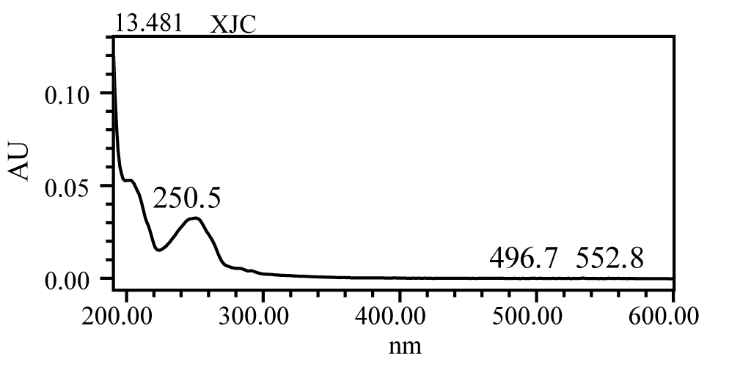


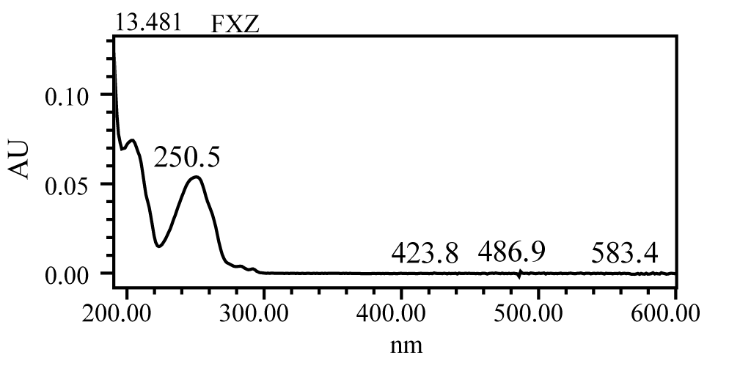


Peak 8:


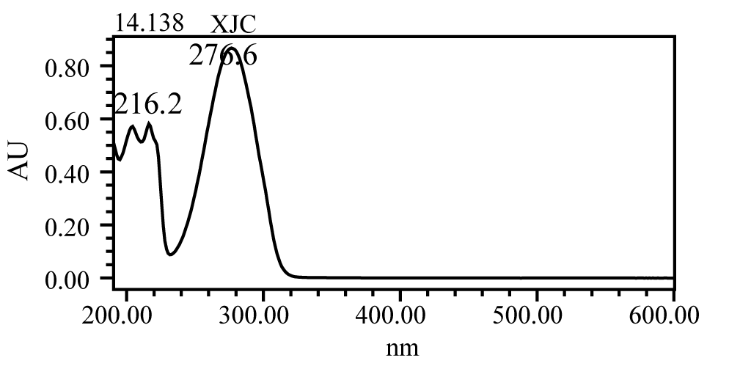


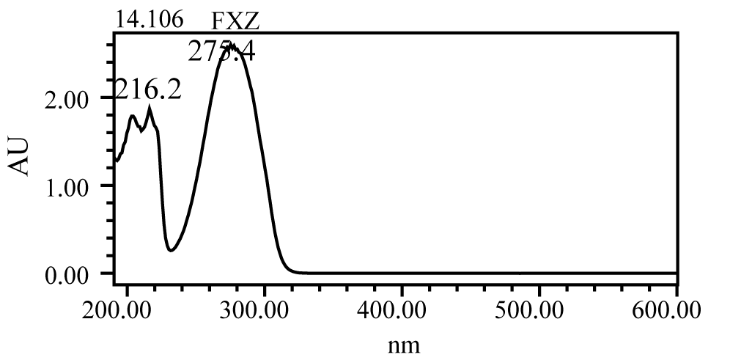


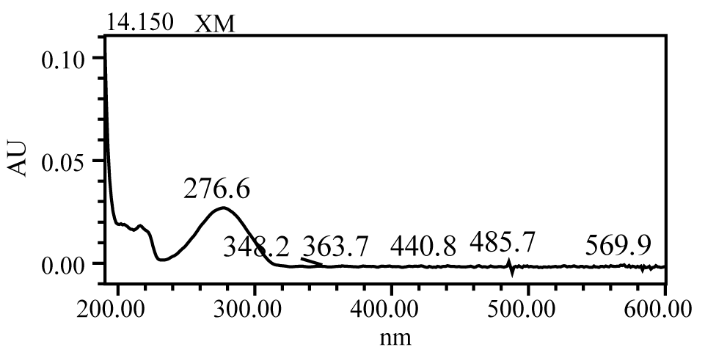


Peak 9:


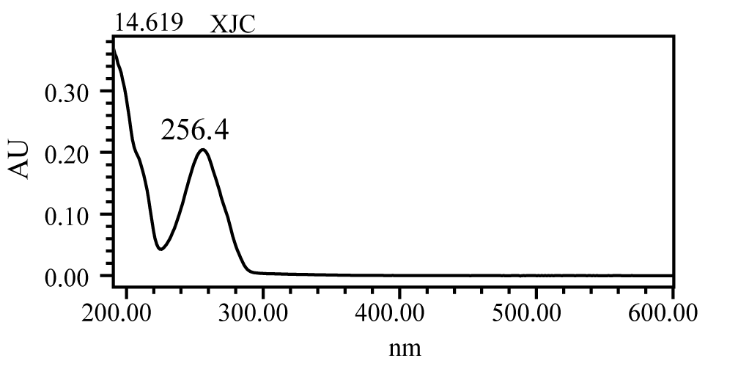

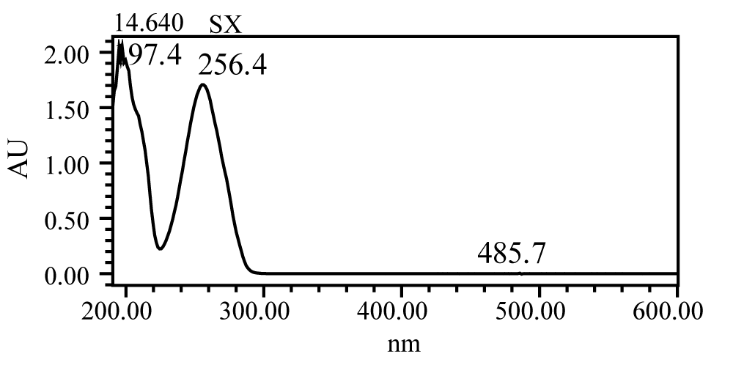


Peak 10:


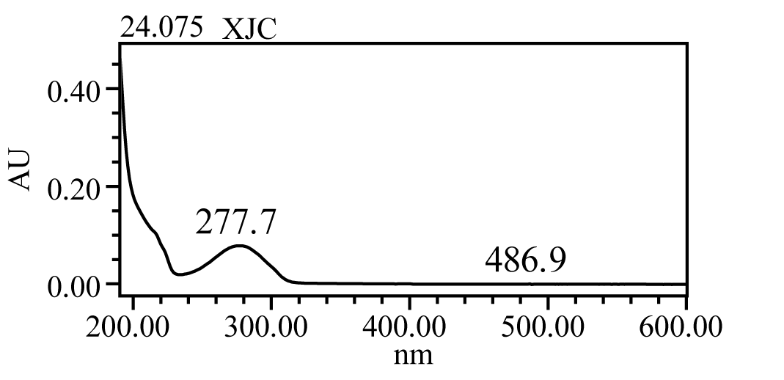

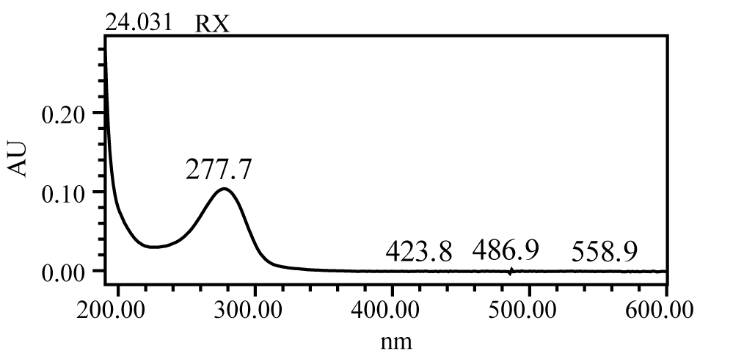


Peak 11:


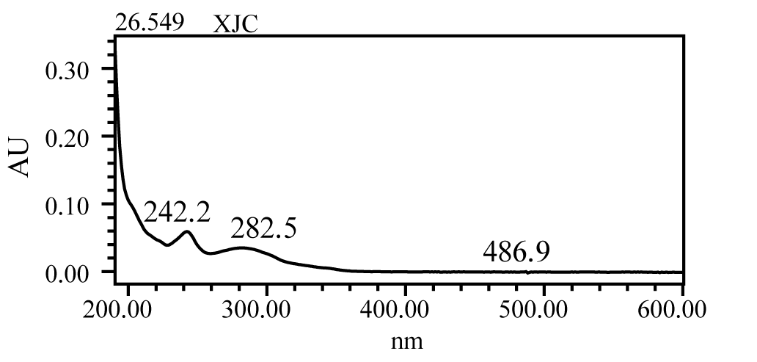

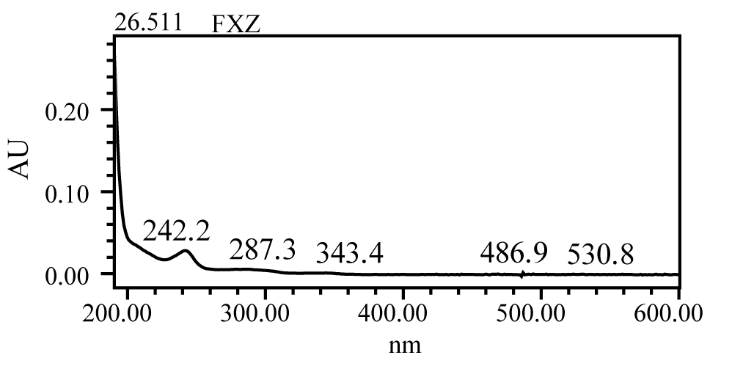


Peak 12:


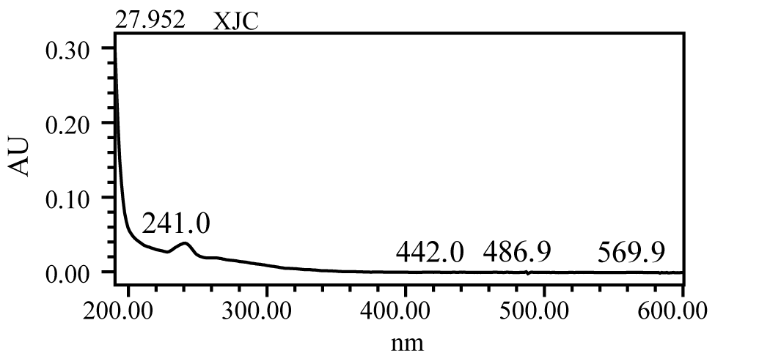

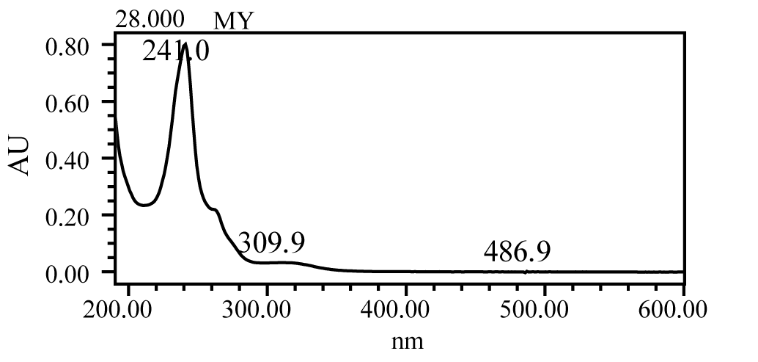


Peak 13:


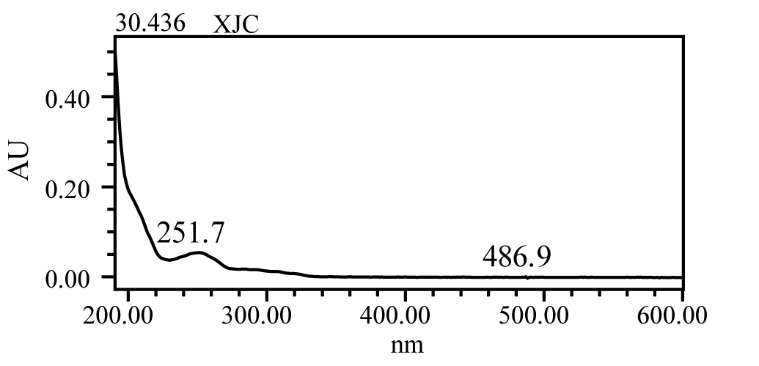

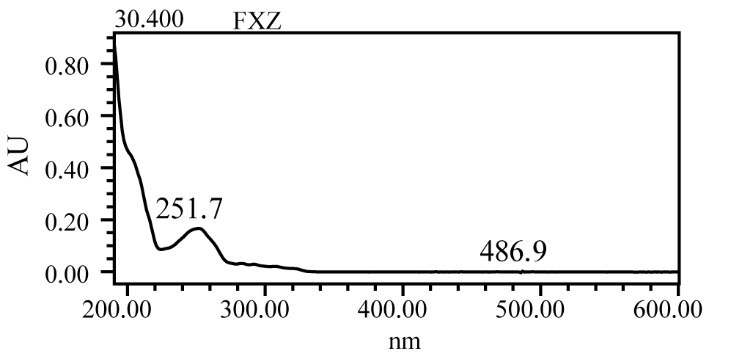


Peak 14:


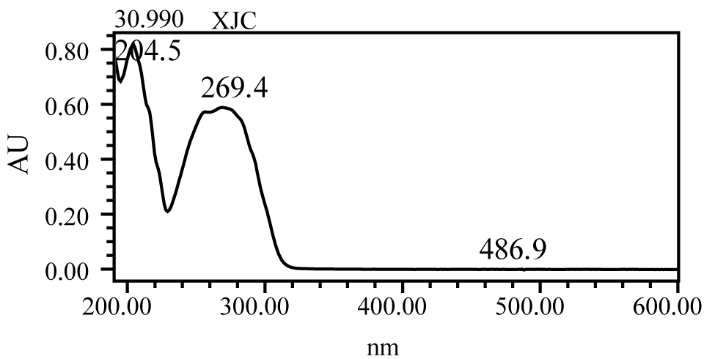

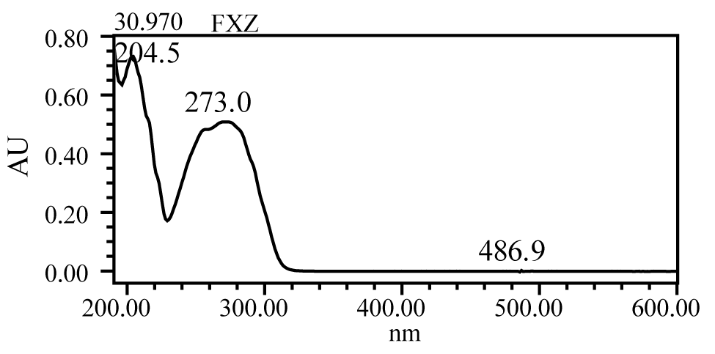


Peak 15:


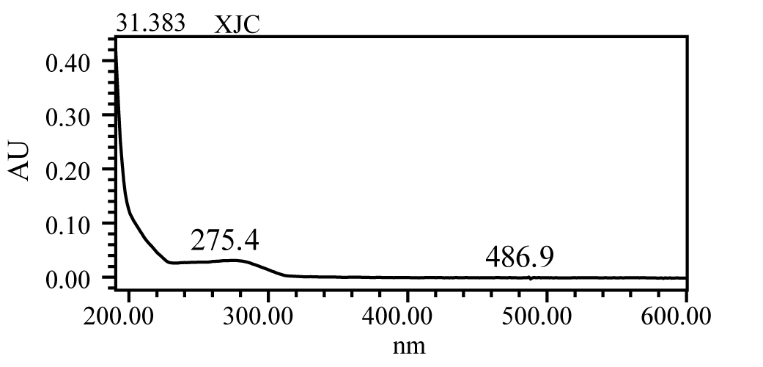

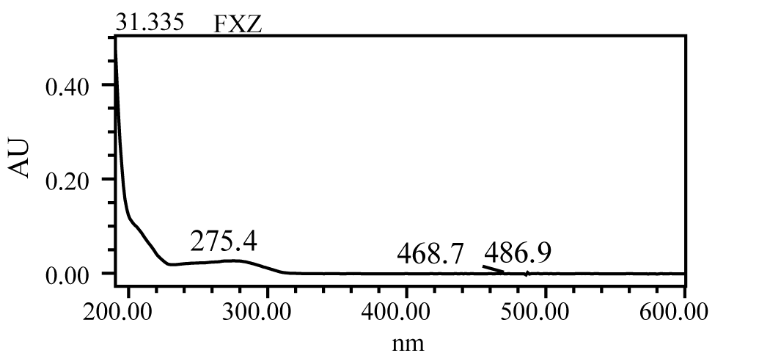

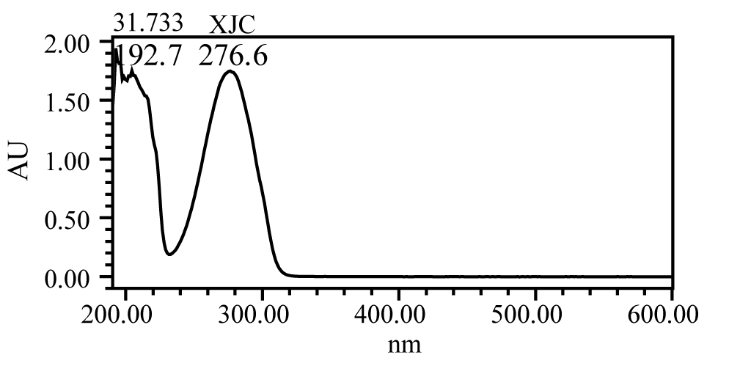

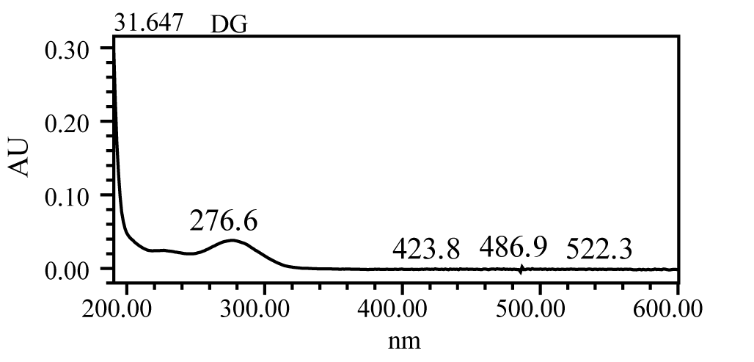


Peak 16:


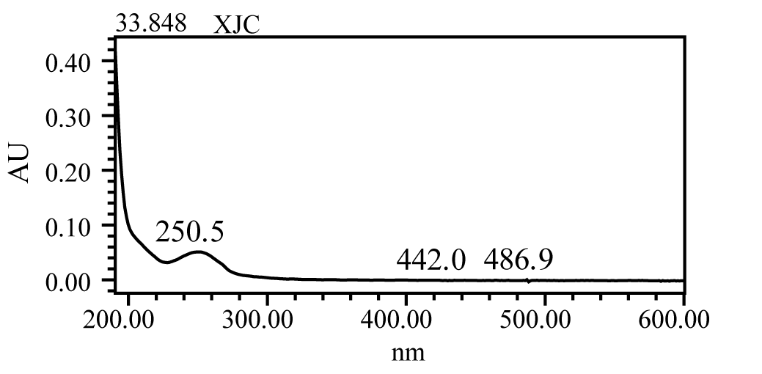

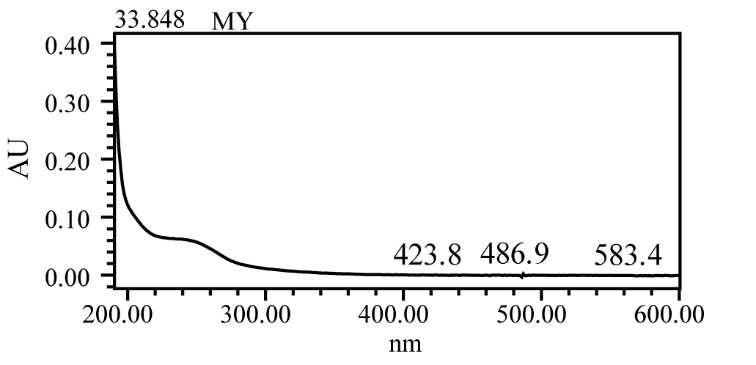

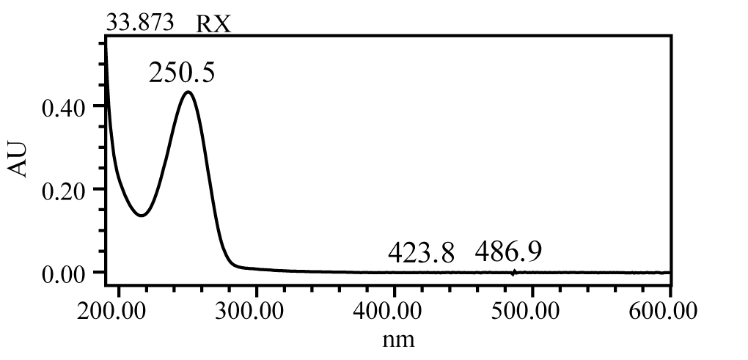


Peak 17:


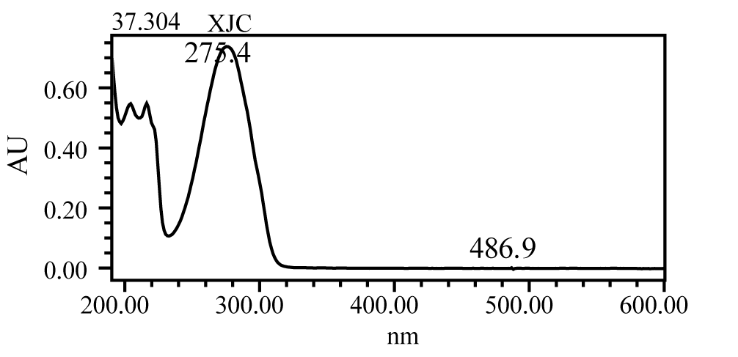

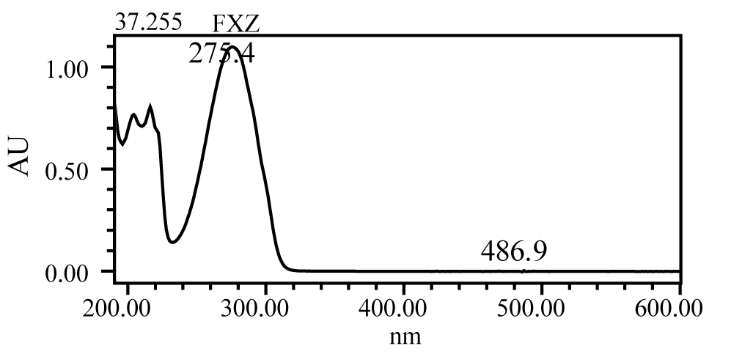


Peak 18:


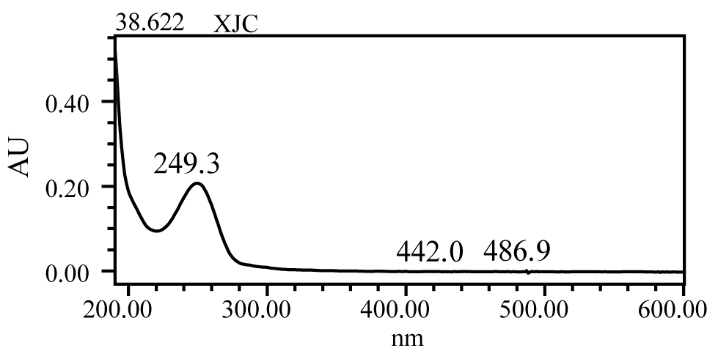

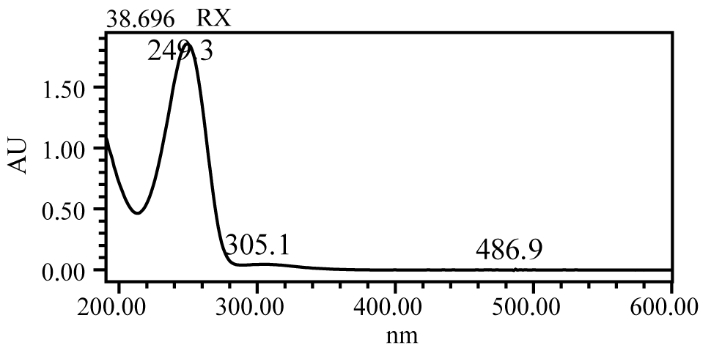

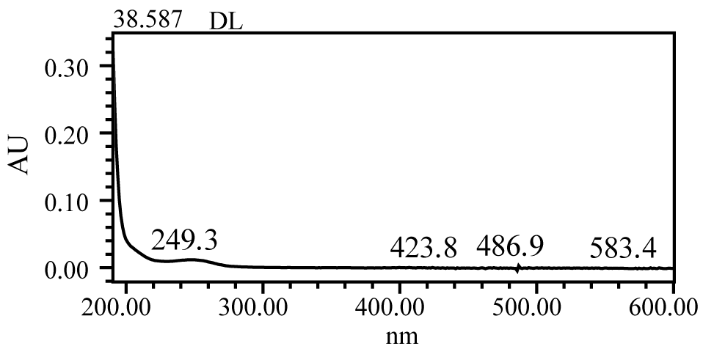


Peak 19:


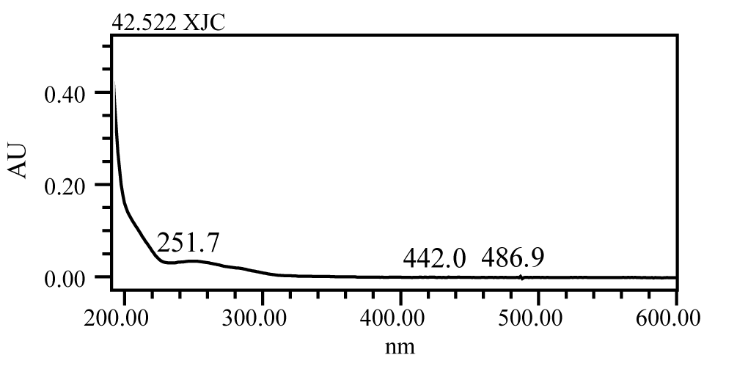

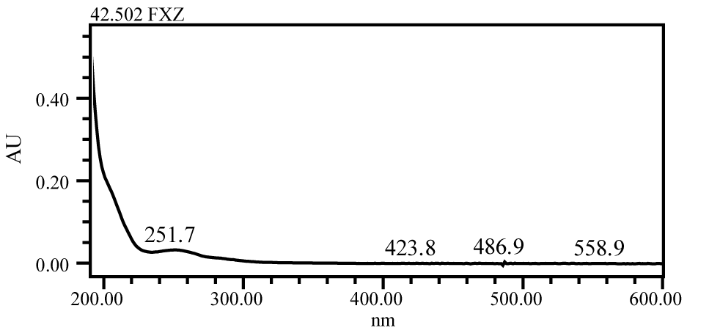

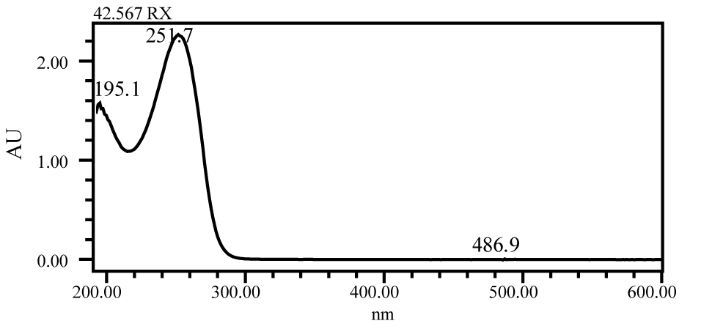


Peak 20:


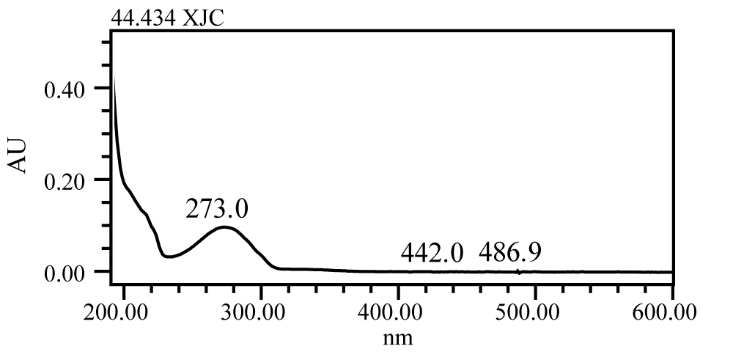

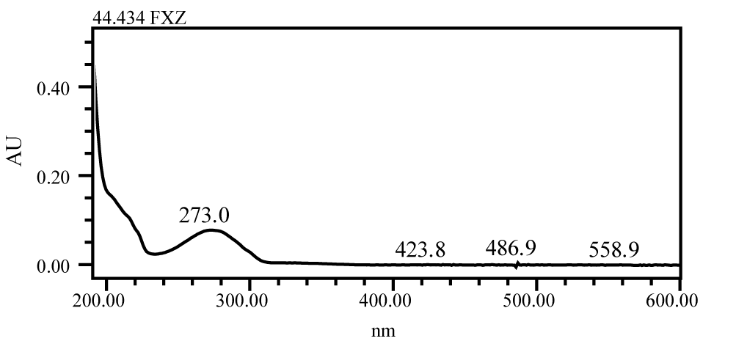


Peak 21:


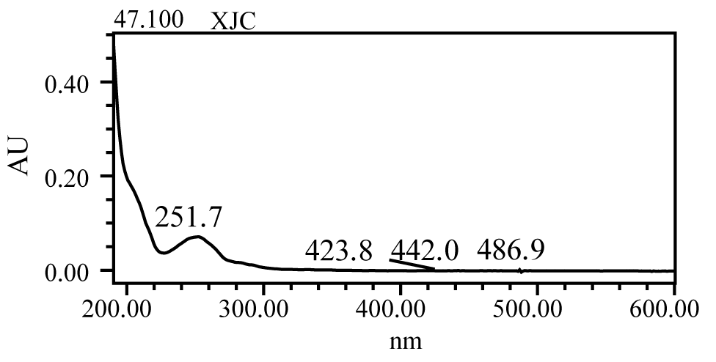

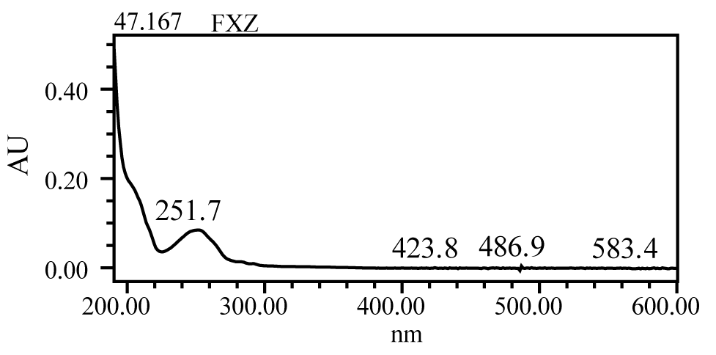


Peak 22:


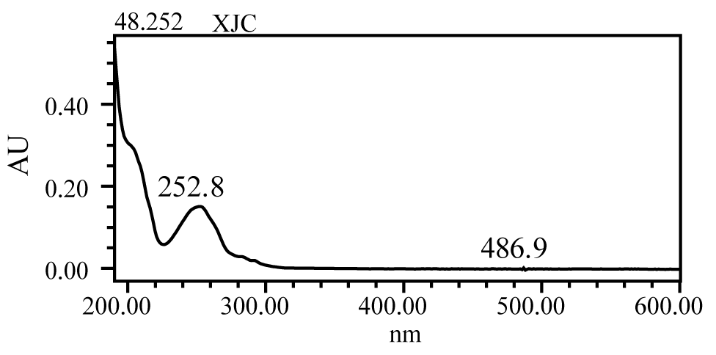

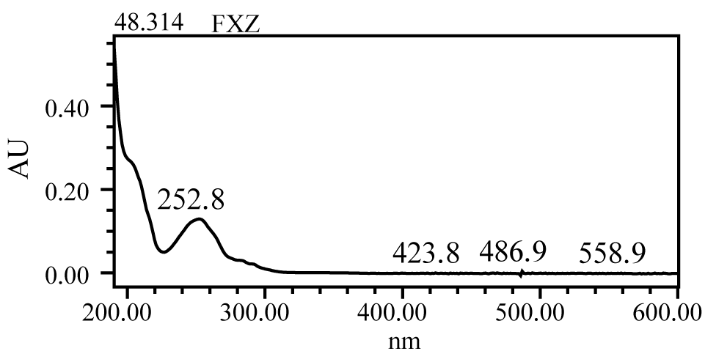


Peak 23:


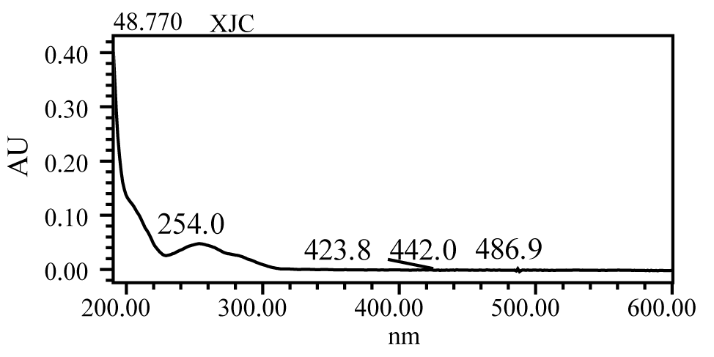

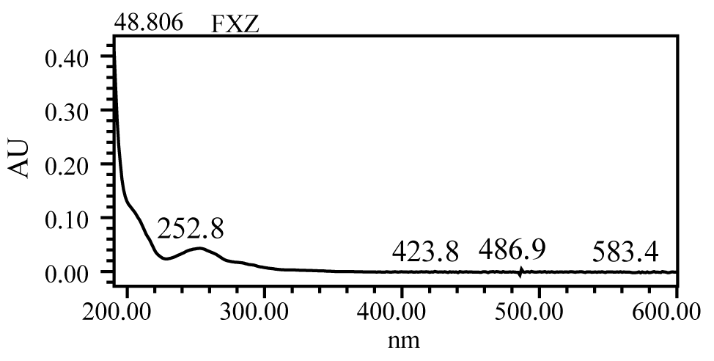


Peak 24:


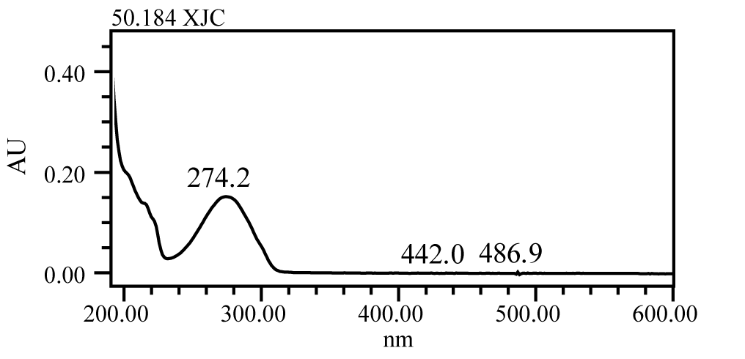

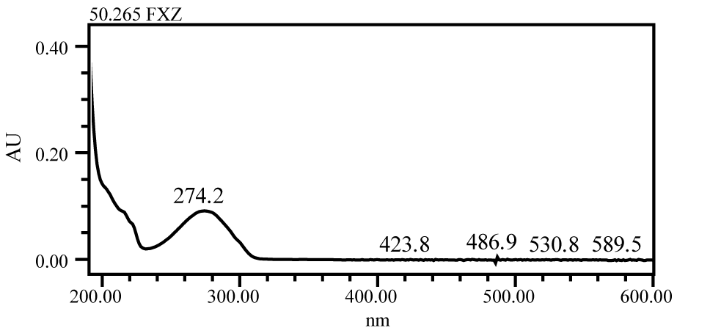


Peak 25:


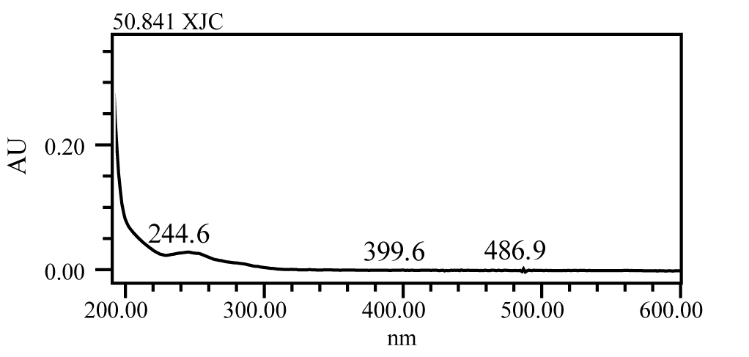

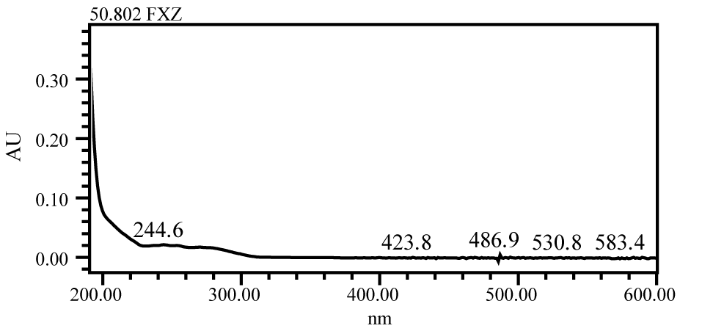


**Fig. S4** Absorption spectra of chromatographic peaks in XJC and herb materials fingerprints.
